# PROMER technology: A new real-time PCR tool enabling multiplex detection of point mutation with high specificity and sensitivity

**DOI:** 10.1093/biomethods/bpae041

**Published:** 2024-06-04

**Authors:** Hwanhee Nam, Esder Lee, Hichang Yang, Kyeyoon Lee, Taeho Kwak, Dain Kim, Hyemin Kim, Mihwa Yang, Younjoo Yang, Seungwan Son, Young-Hyean Nam, Il Minn

**Affiliations:** Institute for NanoBioTechnology, Johns Hopkins University, Baltimore, MD 21218, United States; NuriBio Co., Ltd, Anyang-si, Gyeonggi-Do, 14058, Republic of Korea; NuriBio Co., Ltd, Anyang-si, Gyeonggi-Do, 14058, Republic of Korea; NuriBio Co., Ltd, Anyang-si, Gyeonggi-Do, 14058, Republic of Korea; NuriBio Co., Ltd, Anyang-si, Gyeonggi-Do, 14058, Republic of Korea; NuriBio Co., Ltd, Anyang-si, Gyeonggi-Do, 14058, Republic of Korea; NuriBio Co., Ltd, Anyang-si, Gyeonggi-Do, 14058, Republic of Korea; NuriBio Co., Ltd, Anyang-si, Gyeonggi-Do, 14058, Republic of Korea; NuriBio Co., Ltd, Anyang-si, Gyeonggi-Do, 14058, Republic of Korea; NuriBio Co., Ltd, Anyang-si, Gyeonggi-Do, 14058, Republic of Korea; NuriBio Co., Ltd, Anyang-si, Gyeonggi-Do, 14058, Republic of Korea; Institute for NanoBioTechnology, Johns Hopkins University, Baltimore, MD 21218, United States; Russell H. Morgan Department of Radiology and Radiological Science, Johns Hopkins Medical Institutions, Baltimore, MD 21287, United States

**Keywords:** real-time PCR, circulating tumor DNA, cell-free DNA, KRAS mutations, liquid biopsy

## Abstract

Real-time polymerase chain reaction (real-time PCR) is a powerful tool for the precise quantification of nucleic acids in various applications. In cancer management, the monitoring of circulating tumor DNA (ctDNA) from liquid biopsies can provide valuable information for precision care, including treatment selection and monitoring, prognosis, and early detection. However, the rare and heterogeneous nature of ctDNA has made its precise detection and quantification challenging, particularly for ctDNA containing hotspot mutations. We have developed a new real-time PCR tool, PROMER technology, which enables the precise and sensitive detection of ctDNA containing cancer-driven single-point mutations. The PROMER functions as both a PRObe and priMER, providing enhanced detection specificity. We validated PROMER technology using synthetic templates with known KRAS point mutations and demonstrated its sensitivity and linearity of quantification. Using genomic DNA from human cancer cells with mutant and wild-type KRAS, we confirmed that PROMER PCR can detect mutant DNA. Furthermore, we demonstrated the ability of PROMER technology to efficiently detect mutation-carrying ctDNA from the plasma of mice with human cancers. Our results suggest that PROMER technology represents a promising new tool for the precise detection and quantification of DNA containing point mutations in the presence of a large excess of wild-type counterpart.

## Introduction

Cell-free DNA (cfDNA) is a mixture of DNA fragments found in bodily fluids, originating from host cells in the process of their turnover [1, 2]. cfDNA can contain disease-specific DNA from the host, such as circulating tumor DNA (ctDNA) in the case of cancer patients [3]. Real-time monitoring and analysis of ctDNA has yielded valuable insights for personalized cancer care, including early detection [4–7], assessment of treatment efficacy [8, 9], detection of minimal residual disease [10–13], and identification of drug targets [14, 15] or resistance [16, 17]. ctDNA contains informative features such as genetic variation, copy number variation, fragmentation and rearrangement, and unique epigenetic aberrations. Among these characteristics, cancer-specific point mutations are the first and most used for approved companion diagnostics, such as Epidermal Growth Factor Receptor for non-small cell lung cancer and PIK3CA for breast cancer [5, 11, 18, 19]. Other targets, including ALK, ATM, BRACA1/2, BRAF, KRAS, MET, RET, and ROS1 are undergoing clinical evaluation [20]. Some studies examined a number of known mutations to establish a threshold for tumor detectability from single patient [11, 21, 22]. Together, ctDNA presents promising features for precision health applications.

Despite its potential, the precise and quantitative detection of ctDNA from cfDNA in plasma remains a challenging task. While the concentration of cfDNA in the plasma of cancer patients is higher than that of healthy individuals, the proportion of ctDNA [variant allele frequency (VAF)] in the isolated cfDNA varies significantly for different types of cancer and among patients with the same cancer [23, 24]. ctDNA is believed to be released through the apoptotic or necrotic turnover of cancer cells. However, the rate of cell death varies among different types of cancer, and the background levels of cfDNA originating from hematopoietic cells also vary among individuals [25, 26]. Intra-patient tumor heterogeneity can also complicate the detection of mutant-harboring ctDNA, as its quantity can be extremely low or it may exist in multiple forms of mutation [27, 28]. Furthermore, cfDNA has a rapid turnover, with a reported half-life ranging from 16 to 150 min [23, 29]. These characteristics necessitate the development of fast, highly sensitive, and precise detection tools for the analysis of ctDNA, particularly for the identification of cancer-specific point mutations.

Sequencing, quantitative polymerase chain reaction (PCR), and mass spectrometry have been the techniques widely used for the detection of ctDNA from patients’ liquid biopsies. Each technique offers specialized and in-depth analysis based on its unique principle. Whole-genome or whole-exome sequencing via next-generation sequencing (NGS) can provide comprehensive information on genetic variations [30, 31]. However, the low sensitivity of this approach makes it unsuitable for detecting rare ctDNA with <0.1% VAF. Additionally, NGS is not cost- or time-effective for clinical scenarios requiring rapid analysis. Several targeted sequencing approaches have been developed to investigate only certain loci of interest. These include tagged-amplicon deep sequencing (Tam-Seq) [32], Safe-Sequencing System (Safe-SeqS) [33], simple, multiplexed, PCR-based barcoding of DNA for sensitive mutation detection using sequencing (SiMSen-seq) [34], and cancer personalized profiling by deep sequencing (CAPP-Seq) [21]. PCR-based assays generally achieve enhanced sensitivity and precise quantification. Digital PCR technology such as droplet digital PCR (ddPCR) and BEAMing (beads emulsion amplification and magnetics) have been developed for ctDNA detection [35]. Real-time PCR provides a cost-effective, sensitive, and fast tool for ctDNA detection, especially for mutations with low allele frequency. Advanced real-time PCR such as allele-specific amplification (AS-PCR) [36] and co-amplification at lower denaturation temperature (COLD-PCR) [37] have been developed. Despite these technical advancements in improving detection efficacy and lowering error rates, continued research in ctDNA detection technology for diversified and improved performance is warranted for faster clinical implementation of ctDNA.

In this article, we present a new real-time PCR tool, named as PROMER technology [38], which has been developed for the sensitive and precise detection of small variations in DNA, such as single-point mutations, within a mixture of nucleic acids. We have validated the efficacy of PROMER technology using both synthetic DNA templates and cancer genomic DNA. Additionally, we have tested the technology in a multiplexed assay format. Finally, we have evaluated its capability to detect ctDNA in the blood of cancer-bearing animal models.

## Material and methods

### Reagents for PCR

The KRAS synthetic templates (Supplementary Table S1), PROMERs, and primers (Supplementary Table S2) were custom synthesized by Integrated DNA Technologies (Newark, NJ). KRAS TaqMan^®^ Mutation Detection Assays (Assay IDs: Hs00000113_mu, Hs00000113_mu, Hs00000117_mu, Hs00000119_mu, Hs00000121_mu, Hs00000123_mu, Hs00000125_mu, Hs00000131_mu, Hs00000137_mu, and Hs00000139_mu) were purchased from Applied Biosystems (Waltham, MA, USA). RNase H2 was purchased from BioAssay Co. Ltd (Daejeon, South Korea) or custom produced. Briefly, cDNA of RNase H2 (*pyrococcus furiosus*, GenBank: CP023154.1) was cloned into pET-28a plasmid and transfected into BL21(DE3)pLysS strain. The expression of the RNase H2 was induced with IPTG and purified using Ni-NTA affinity chromatography (ThermoFisher Scientific, Waltham, MA, USA). 5x Apta Taq DNA Master was purchased from Roche (Basel, Switzerland).

### Cell lines and genomic DNA preparation

The cell lines listed in Supplementary Table S3 were purchased from the American Type Culture Collection (ATCC, Manassas, Virginia) and were maintained according to the provider’s instructions. Genomic DNA from the cell lines was extracted using the Quick-DNA Miniprep Plus kit (Zymo Research, Irvine, CA, USA) according to the manufacturer’s instructions. Isolated genomic DNA was quantified using a NanoDrop™ Lite (ThermoFisher Scientific, Waltham, MA, USA).

### Animals study

The animal experiments were conducted in accordance with a protocol approved by the Johns Hopkins Animal Care and Use Committee, and in compliance with the regulations of the Animal Welfare Act and Public Health Service (PHS) Policy. Johns Hopkins University has an approved PHS assurance. NSG (NOD/SCID/IL2Rγnull) mice were obtained from the Animal Resources Core of the Sydney Kimmel Comprehensive Cancer Center at Johns Hopkins University. NSG mice were administered with 1 million cells on their lower left flank. Tumor sizes were recorded twice a week. The animals were observed daily for changes in weight and any abnormalities. Animals were euthanized using a CO_2_ chamber, and whole blood was collected in 1.7 ml tube supplemented with 180 µl of acid citrate dextrose (ACD) solution (Becton Dickinson, Franklin Lakes, NJ, USA) for the isolation of cfDNA. The cfDNA was isolated using the MagMax Cell-Free DNA Isolation kit (Applied Biosystems, Waltham, MA, USA). The length and quantity of the isolated cfDNA were measured using the Agilent 2100 BioAnalyzer (Agilent, Santa Clara, CA, USA) at the Johns Hopkins Sidney Kimmel Comprehensive Cancer Center Experimental and Computational Genomics Core.

### PCR reactions

PCR reactions were performed using QuantStudio 3, QuantStudio 5, QuantStuido 12K Flex (Applied Biosystems, Waltham, MA, USA), or CFX96 Touch Real-Time PCR Detection System (Bio-Rad, Hercules, CA, USA). Detailed PCR setup and thermal-cycling conditions are listed in Supplementary Table S4.

## Results

### PROMER technology for precise and sensitive detection of single-point mutation

**Figure 1.**
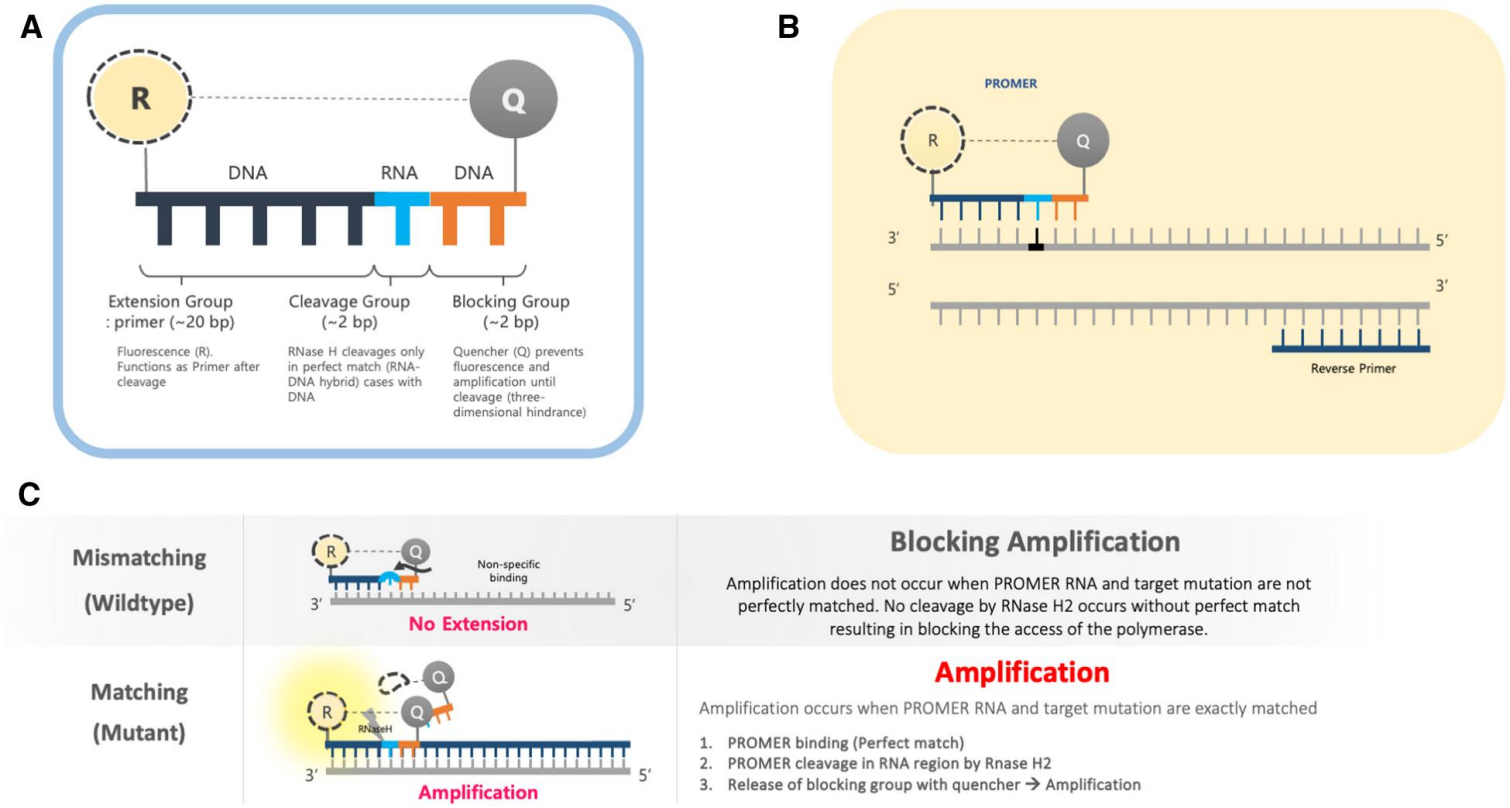
Schematic representation of the PROMER technology. (A) Key components of the PROMER system. (B) PROMER PCR requires the use of one PROMER and one Primer for amplification. (C) Mechanism of mutant-specific amplification using PROMER technology. R represents reporter fluorescent dyes (FAM, HEX, or Cy5), while Q represents the quencher molecule.

We developed a new real-time PCR tool named PROMER (a combination of PRObe and priMER) technology designed for the precise and sensitive detection of single-point mutation. The PROMER is composed of a DNA–RNA–DNA hybrid oligonucleotide that has a fluorophore attached to its 5′-end and a quencher attached to its 3′-end (Fig. 1A). The DNA bases at the 5′-end of the PROMER serve as an extension group that acts as a 5′-primer for the PCR reaction. The cleavage group, which consists of 1 or 2 RNA bases, provides detection specificity by enabling a type 2 Ribonuclease H (RNase H2) to cut the phosphodiester bond of RNA in the DNA: RNA double strand, leaving a 3′-hydroxyl group. This cleavage only takes place when the DNA: RNA base pairing is an exact match [39, 40]. The principle of integrating RNase H2 into PCR (rhPCR) has been previously explored and demonstrated to enhance the specificity of PCR detection by mitigating off-target amplifications [41]. This study utilized two sets of blocked primers, which exhibited reduced extension efficiency in the absence of primer cleavage by RNase H2. We took the same principle of the match-specific cleavage of RNA: DNA hybrid pairing by RNase H2 for PROMER technology and developed it into more versatile tool capable of detecting rare and shorter targets in the mixture of genomic DNA with a high degree of precision. The blocking group, 3′-end DNA bases of the PROMER serves two functions: it quenches the fluorophore attached to the extension group and it creates a steric hindrance that prevents DNA polymerase II from elongating the strand if cleavage does not occur. PROMER technology only requires a single primer and a PROMER, while a conventional probe-based PCR (such as TaqMan™) needs an additional pair of primers along with the specific probe (Fig. 1B). If there is a mismatch in the cleavage group (e.g. wild-type allele with the mutant-matching PROMER), no cleavage occurs resulting in no amplification from the PROMER PCR (Fig. 1C). However, if the mutant allele is present, the PROMER PCR will generate real-time amplification (Fig. 1C).

### Validation of PROMER technology using synthetic templates of KRAS mutations

To validate PROMER technology’s ability to detect point mutations accurately, we chose the human KRAS gene as our test subject. KRAS has various mutations at its 12th and 13th Glycine residues that require specific probes to differentiate them [42], which would determine the therapeutic regimen developed for the specific mutations. We synthesized total of 15 KRAS DNA templates including 13 known point mutations of G12C/S/R (at the 34th base), G12/V/D/A (at the 35th base), G13C/S/R (at the 37th base), G13/V/D/A (at the 38th base), Q61H (at the 183rd base), and two wild-types (Supplementary Table S1). We also created the PROMERs specific to these mutations, with selected fluorophore conjugated at its 5′-end and the quencher at its 3′-end (Supplementary Table S2). Serially diluted synthetic templates ranging from 1 × 10^5^ copies to one copy were tested for the detection limit of each PROMER (Table 1). The PROMER PCR successfully detected one copy of the designated synthetic template for G12C/S/R/V/A, G13C/V/D, and Q61H (Table 1), while PROMERs for G12D and G13S/R/A detected 10 copies of the template (Table 1). There was no non-specific amplification in the no template control (NTC), indicating that the PROMER PCR can specifically detect low-copy number targets.

**Table 1.** PROMER PCR detects low level of synthetic template with KRAS mutations.

	Template copy^a^	Ct mean		Template copy^a^	Ct mean
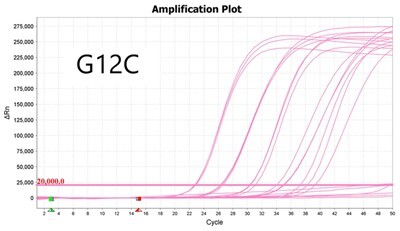	1 × 10^5^	22.756	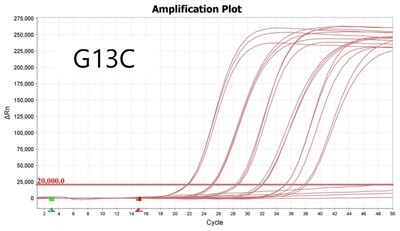	1 × 10^5^	21.720
	1 × 10^4^	26.377		1 × 10^4^	25.094
	1 × 10^3^	30.558		1 × 10^3^	28.494
	1 × 10^2^	34.623		1 × 10^2^	31.823
	1 × 10^1^	37.721		1 × 10^1^	35.513
	1 × 10^0^	44.193		1 × 10^0^	37.888
	NTC	ND		NTC	ND
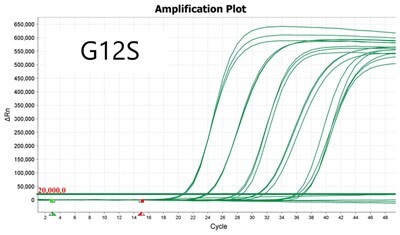	1 × 10^5^	20.668	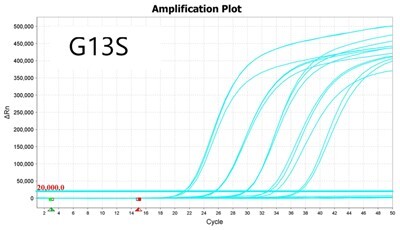	1 × 10^5^	21.401
	1 × 10^4^	23.779		1 × 10^4^	25.562
	1 × 10^3^	28.108		1 × 10^3^	29.796
	1 × 10^2^	30.961		1 × 10^2^	33.184
	1 × 10^1^	36.509		1 × 10^1^	37.076
	1 × 10^0^	36.087		1 × 10^0^	ND
	NTC	ND		NTC	ND
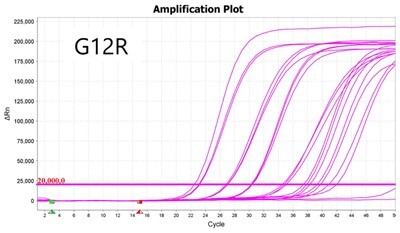	1 × 10^5^	22.896	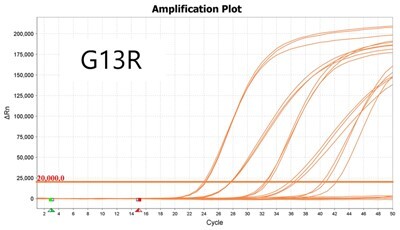	1 × 10^5^	24.101
	1 × 10^4^	26.941		1 × 10^4^	27.697
	1 × 10^3^	30.319		1 × 10^3^	32.622
	1 × 10^2^	34.751		1 × 10^2^	36.273
	1 × 10^1^	37.854		1 × 10^1^	41.067
	1 × 10^0^	40.883		1 × 10^0^	ND
	NTC	ND		NTC	ND
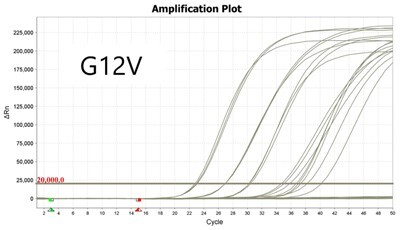	1 × 10^5^	22.925	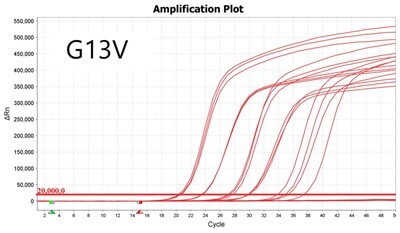	1 × 10^5^	20.571
	1 × 10^4^	26.942		1 × 10^4^	23.565
	1 × 10^3^	30.255		1 × 10^3^	27.439
	1 × 10^2^	35.160		1 × 10^2^	29.888
	1 × 10^1^	37.028		1 × 10^1^	35.469
	1 × 10^0^	39.158		1 × 10^0^	35.328
	NTC	ND		NTC	ND
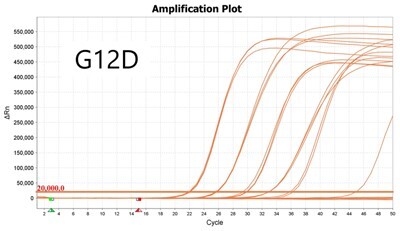	1 × 10^5^	22.004	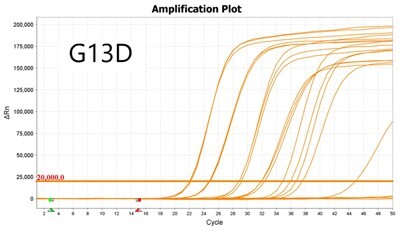	1 × 10^5^	22.090
	1 × 10^4^	25.714		1 × 10^4^	24.814
	1 × 10^3^	29.711		1 × 10^3^	28.947
	1 × 10^2^	32.924		1 × 10^2^	32.103
	1 × 10^1^	36.061		1 × 10^1^	35.849
	1 × 10^0^	ND		1 × 10^0^	37.582
	NTC	ND		NTC	ND
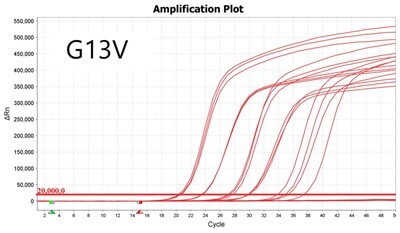 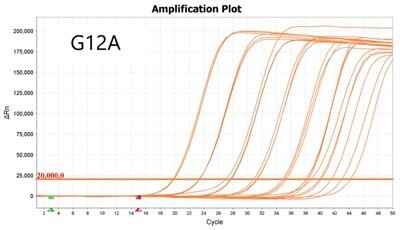	1 × 10^5^	22.980	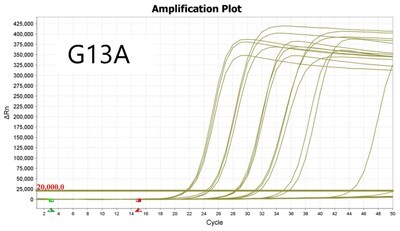	1 × 10^5^	21.685
	1 × 10^4^	28.599		1 × 10^4^	24.627
	1 × 10^3^	31.224		1 × 10^3^	28.454
	1 × 10^2^	35.066		1 × 10^2^	31.271
	1 × 10^1^	40.726		1 × 10^1^	38.292
	1 × 10^0^	41.562		1 × 10^0^	ND
	NTC	ND		NTC	ND
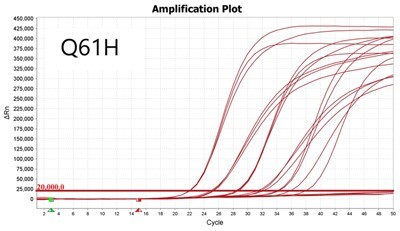	1 × 10^5^	22.018	#: number NTC: No Template Control ND: Not Detected		
	1 × 10^4^	25.129			
	1 × 10^3^	28.554			
	1 × 10^2^	31.742			
	1 × 10^1^	35.451			
	1 × 10^0^	35.841			
	NTC	ND			

Each reaction was performed in triplicates.

a Number.

ND, not detected.

### Validation of PROMER technology using genomic DNA from human cancer cell lines with KRAS mutations

We conducted further validation of the PROMER technology to assess its ability to detect human genomic DNA with KRAS mutations and compared its efficacy to that of synthetic templates. Human cancer cell lines with KRAS mutations of G12C/S/R/V/D/A, G13C/D, and Q61H were obtained from ATCC (Supplementary Table S3) and their genomic DNAs were prepared for use as test templates. Serial dilutions of the genomic DNAs were tested using PROMER PCR, with copy numbers ranging from 1 × 10^4^ to 1 × 10° for each genotype, as well as NTC (Supplementary Table S4). The Profit analysis was employed to evaluate the detectability of the PROMER PCR, with a detection probability of 93% or higher considered as a detectable copy number (Supplementary Table S5) [43]. The linearity between the tested copy numbers and the average Ct values was also determined. All tested PROMERS were able to detect one copy of each genomic DNA (Table 2 and Supplementary Table S6), with a coefficient of determination (*R*^2^) value of ≥0.99 DNA (Table 2 and Supplementary Table S7).

**Table 2.** Detection limit and linearity of PROMER PCR for genomic DNAs with KRAS mutation.

Genotype	Cell line	Detection Limit (Copy number)	*R* ^2^
G12C	MIA PaCa-2	1	0.994
G12S	A549	1	0.993
G12R	MDA-MB-134VI	1	0.992
G12V	SW620	1	0.999
G12D	SNU-C2B	1	0.990
G12A	SW1116	1	0.997
G13C	NCI-H1734	1	0.996
G13D	HCT-15	1	0.994
Q61H	NCI-H460	1	0.994

Data used for the table are presented in Supplementary Table S5.

We also determined the detection specificity of PROMER PCR for KRAS mutations. Mutant genomic DNAs of 1 ×10^3^, 1 × 10^2^, 1 × 10^1^, 1 × 10^0^, and 0 copies in the presence of 1 × 10^4^ copies of KRAS wild-type genomic DNA from NCI-H1975 cells were tested as templates. We compared the detection capability of PROMER PCR with commercial TaqMan assays for KRAS single-point mutation. Results showed that PROMER PCR exhibited comparable (G12C/S/A, G13C/D, and Q61H) or superior (G12S/R/V) sensitivity to the commercial probe-based PCR for the tested mutations (Table 3 and Supplementary Table S6).

**Table 3.** Detection Specificity of PROMER and TaqMan PCR for genomic DNAs with KRAS mutation.

Genotype	Cell line	Detection limit (copy number) PROMER PCR	Detection limit (copy number) TaqMan PCR
G12C	MIA PaCa-2	1	1
G12S	A549	10	10
G12R	MDA-MB-134VI	1	NT
G12V	SW620	1	10
G12D	SNU-C2B	1	10
G12A	SW1116	10	10
G13C	NCI-H1734	1	1
G13D	HCT-15	10	10
Q61H	NCI-H460	1	1

All reactions were performed in the presence of 10^4^ copies of the wild-type genomic DNA. Data used for the table are presented in Supplementary Table S6. NT, not tested.

We conducted a comprehensive examination of all KRAS G12 variants (G12C/S/R/V/D/A) to assess their cross-reactivity with other G12 types. We tested PROMERS for G12 variants (*n* = 20) against genomic DNAs of varying quantities (15, 30, 45, and 60 ng) prepared from cell lines representing each mutant type and the wild-type (Supplementary Table S3). All PROMERs demonstrated robust PCR amplification with the corresponding genomic DNA (Table 4). In contrast, when tested against non-matching genomic DNA, the PROMERs exhibited negligible PCR amplification (Table 4 and Supplementary Table S8). By setting a Ct cut-off value of 38, we observed no non-specific amplification for all G12 PROMERs. These results further confirm the specificity of our PROMER technology.

**Table 4.** Detection Specificity of G12 PROMERs for genomic DNAs with other G12 mutations.

PROMER (Cell line)	Target Templates						Template amount
	G12C	G12S	G12R	G12V	G12D	G12A	
Wild-type (HEK293)	0/20	0/20	0/20	1/20	1/20	0/20	15 ng
	0/20	2/20	0/20	0/20	0/20	0/20	30 ng
	0/20	5/20	0/20	0/20	1/20	0/20	45 ng
	0/20	1/20	0/20	0/20	3/20	0/20	60 ng
G12C (SW1573)	20/20 (28.50)	0/20	0/20	0/20	0/20	0/20	15 ng
	20/20 (28.00)	0/20	3/20	0/20	0/20	0/20	30 ng
	20/20 (27.63)	0/20	2/20	0/20	0/20	0/20	45 ng
	20/20 (27.39)	0/20	1/20	0/20	0/20	0/20	60 ng
G12S (A549)	0/20	20/20 (27.55)	0/20	0/20	1/20	0/20	15 ng
	0/20	20/20 (26.56)	0/20	0/20	0/20	0/20	30 ng
	0/20	20/20 (25.96)	0/20	0/20	0/20	0/20	45 ng
	0/20	20/20 (25.97)	0/20	0/20	0/20	0/20	60 ng
G12R (MDA-MB-134VI)	0/20	1/20	20/20 (29.68)	0/20	0/20	0/20	15 ng
	0/20	0/20	20/20 (28.71)	0/20	0/20	0/20	30 ng
	0/20	0/20	20/20 (28.03)	0/20	0/20	0/20	45 ng
	0/20	1/20	20/20 (28.50)	0/20	0/20	0/20	60 ng
G12V (SW620)	0/20	0/20	0/20	20/20 (25.50)	0/20	0/20	15 ng
	0/20	0/20	0/20	20/20 (24.69)	0/20	0/20	30 ng
	0/20	0/20	0/20	20/20 (24.28)	1/20	0/20	45 ng
	0/20	0/20	0/20	20/20 (24.03)	0/20	0/20	60 ng
G12D (SNU-C2B)	0/20	0/20	0/20	0/20	20/20 (29.88)	0/20	15 ng
	0/20	0/20	0/20	0/20	20/20 (29.27)	0/20	30 ng
	0/20	0/20	0/20	0/20	20/20 (29.04)	0/20	45 ng
	0/20	0/20	0/20	0/20	20/20 (28.83)	0/20	60 ng
G12A (NCI-H2009)	0/20	0/20	0/20	1/20	1/20	20/20 (30.79)	15 ng
	0/20	0/20	0/20	2/20	1/20	20/20 (30.24)	30 ng
	0/20	1/20	0/20	6/20	0/20	20/20 (29.66)	45 ng
	0/20	0/20	0/20	5/20	2/20	20/20 (28.95)	60 ng

Number represents frequency of amplifications per 20 repeated PCR reactions. Numbers in parenthesis represent the average Ct values for the matching genotypes. Ct values for all non-matching genotypes are greater than 38. Data used for the table are presented in Supplementary Table S8.

### Multiplex detection of the PROMER PCR

Given the high specificity of the PROMER technology, which requires an exact match between the target template and the PROMER for PCR amplification, we hypothesized that PROMER PCR could enable multiplex PCR with mixture of the PRPMERs to detect target DNA in a single well. We designed three unique PROMERs with different fluorophores (HEX, FAM, and Cy5) for this application (Supplementary Table S2). The mixture for three PROMERs targeting KRAS G12C/S/R and G12V/D/A was tested for its ability to specifically detect its target using serially diluted genomic DNA from representative cell lines mixed with 1 × 10^5^ copies of wild-type genomic DNA (Supplementary Table S9). All tested PROMER multiplex PCRs were capable of detecting the designated mutant genomic DNA (Table 5).

**Table 5.** Multiplex PROMER PCR.

PROMER multiplex	Template genotype	Detection limit (copy^a^)	Template genotype	Detection limit
G12C/S/R	G12C	10	G12S	5
	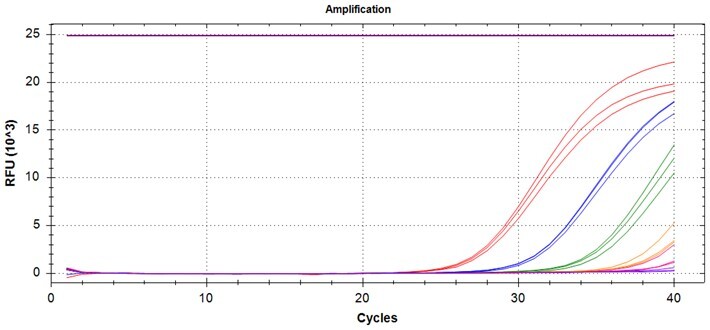		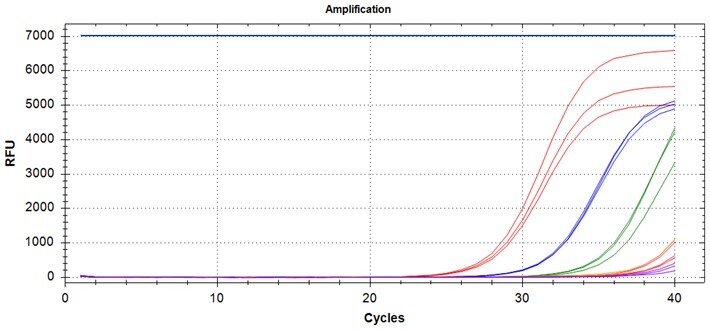	
	G12R	1	Wild-type	ND
	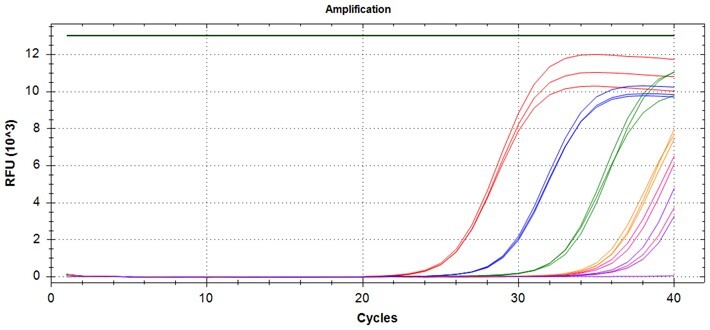		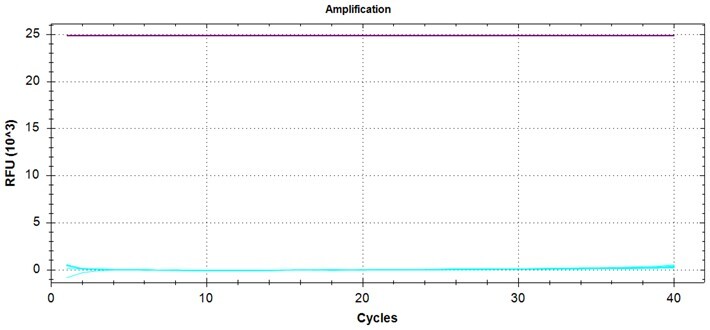	
G12V/D/A	G12V	1	G12D	1
	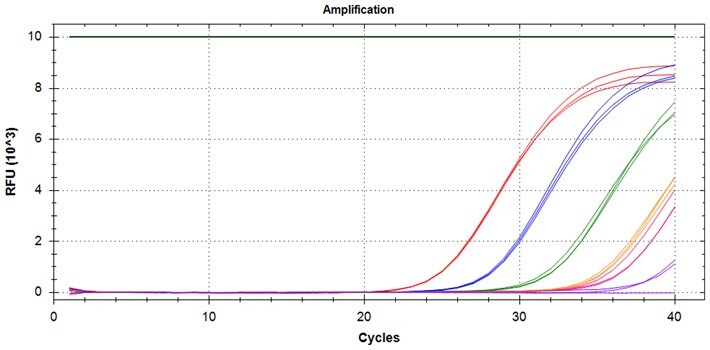		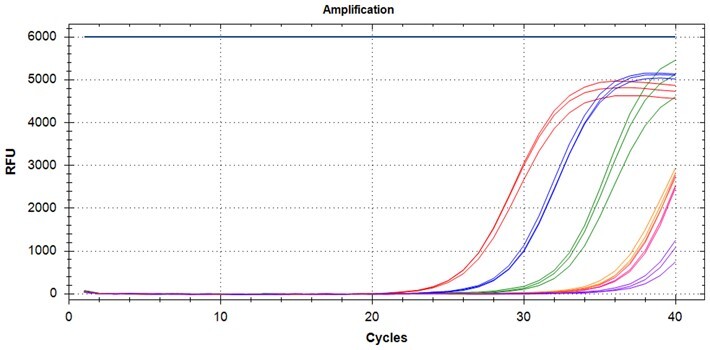	
	G12A	1	Wild-type	ND
	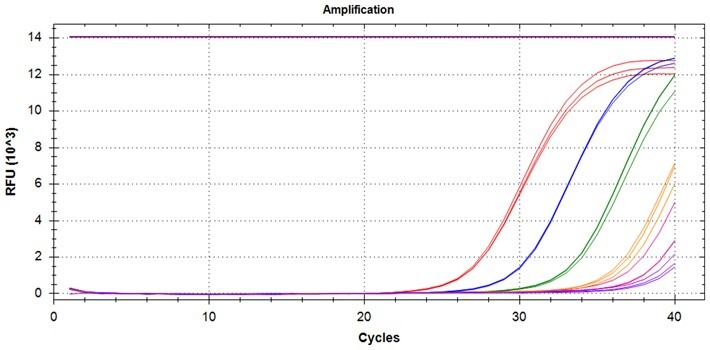		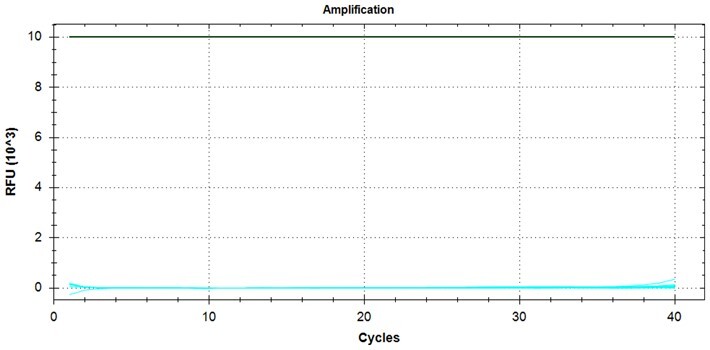	

Mixture for three PROMERs (G12C/S/R or G12V/D/A) were tested in the presence of 10^5^ copies of wild-type genomic DNA. Each reaction was performed in triplicates. a: copy number. Data used for the table are presented in Supplementary Table S9.

### Detection of ctDNA from plasma of tumor-bearing mice using the PROMER PCR

**Figure 2.**
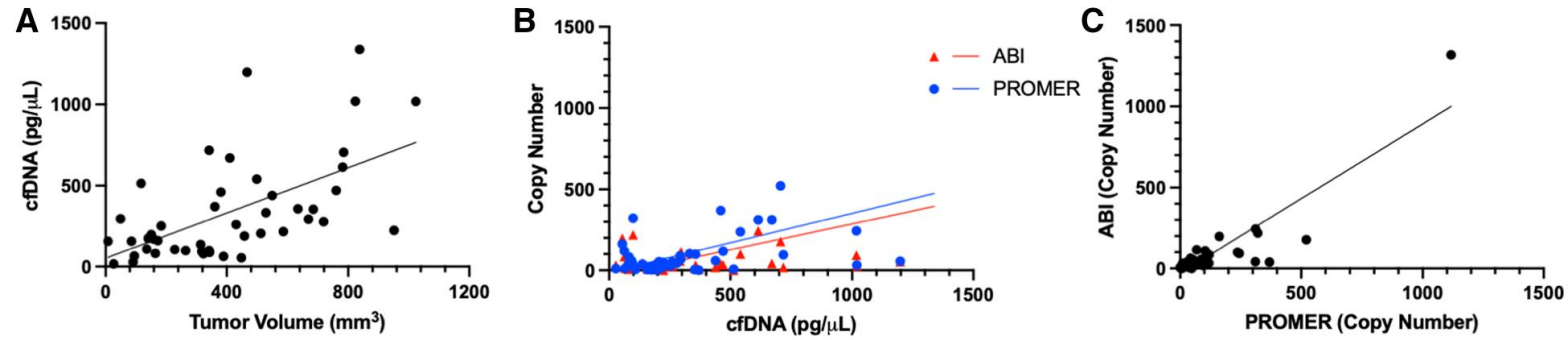
PROMER PCR detects ctDNA from plasma of mice with subcutaneous tumors with a KRAS mutation. (A) Comparison of tumor volumes and the amount of isolated cfDNA. (B) Relationship between the amount of isolated cfDNA and the copy number of detected ctDNA as measured by PROMER PCR and TaqMan PCR. (C) Correlation analysis between ctDNA copy numbers detected by PROMER and TaqMan PCR. Each data point represents a value obtained from a single mouse.

We further sought to examine the PROMER technology for its capability to detect ctDNA in blood plasma originating from the tumor. Subcutaneous (SC) tumors of varying sizes were developed using cancer cell lines for KRAS G12C/S/V/D/A, G13D, and Q61H (Supplementary Table S3) in NSG (NOD/Shi-*scid*/IL-2Rγ^null^) mice. MDA-MB-134VI (G12R) and NCI-H1734 (G13C) did not develop SC tumors in NSG mice. We isolated cfDNA from the peripheral blood of the animals and its quantity and average size of cfDNA was measured using Agilent 2100 Analyzer (Supplementary Fig. S1). The amount of the isolated cfDNA from each model varied but we observed a positive correlation between the tumor volume and the amount of isolated cfDNA (Fig. 2A and Supplementary Table S10). We successfully detected mutation-containing ctDNA from the cfDNA using the PROMER PCR and commercial KRAS detection agent purchased from ABI (Fig. 2B and Supplementary Table S10). The copy numbers were calculated using the standard curves generated with purified genomic DNA from each cell line (Supplementary Table S7). It is important to note that ctDNA quantity can vary depending on tumor type, size, and stage [23, 24, 27, 29]. The observed poor correlation between cfDNA copy number and detected ctDNA (Fig. 2B) may reflect the inherent heterogeneity of ctDNA. Nevertheless, both PROMER and ABI PCRs detected comparable copy numbers from each sample (Fig. 2C and Supplementary Table S10).

## Discussion

In the era of precision medicine, the accurate identification and quantification of rare disease-originated nucleic acids from liquid biopsies have become increasingly important. These nucleic acids can provide valuable information for disease management, including the selection of treatment options, monitoring treatment efficacy, predicting prognosis, and identifying and characterizing acquired drug resistance [5]. However, detecting these disease-specific nucleic acids poses a significant challenge due to their rarity and subtle differences from their wild-type counterparts. High detection sensitivity and specificity are therefore required.

To address this challenge, we present the PROMER technology, a new real-time PCR method capable of precisely detecting rare nucleic acids. The specificity of PROMER technology comes from its unique design of PROMER and the matching sequence-specific cleavage of RNase H2 (Fig. 1). The generation of a PROMER, a DNA–RNA–DNA hybrid specific to its target, requires careful optimization of both its length and the positioning of the hybrid within the PROMER itself. In order to attain high levels of sensitivity and specificity, we performed further optimization in the PCR reaction in conjunction with sequence optimization of the PROMER (Supplementary Table S2). This involved fine-tuning parameters such as the concentration of the PROMER, the annealing temperature, and the extension temperature (Supplementary Table S4). Through our optimization process, we discovered that a higher temperature of 64°C for both annealing and extension steps yielded the highest specificity in detecting mutant templates (Supplementary Table S4). Utilization of a lower temperature for annealing and extension (below 60°C) could result in non-specific PCR reactions with the wild-type template (Supplementary Table S11). In order to facilitate the use of a higher annealing and extension temperature, our design objective for the PROMER was to achieve a melting temperature (TM) within the range of 61–62°C. Our validation data with synthetic templates and genomic DNA from human cancer cell lines with KRAS variant mutations demonstrate the efficiency of PROMER PCR (Tables 1 and 2). Further extensive testing of PROMER PCR with an excess amount of wild-type and genomic DNA of other variants (Table 4) confirms its detection specificity. Verifying the target-specific amplification by PROMER PCR through Sanger sequencing of the amplicons would provide more convincing evidence. However, due to the short length of the PROMER PCR amplicons, Sanger sequencing becomes impractical. This is because the sequencing reads for the first 40–50 base pairs (bp) are often unreliable, and the location of the mutation for our amplicon is within 39 bp from the end of the primer. Furthermore, the absence of PCR amplification of the mutant PROMER PCR with non-matching templates indirectly confirms the specificity of our mutation-specific PCR amplification.

PROMER technology employs a unique approach by using a single primer and a PROMER, which serves dual roles as both a probe and a primer (Fig. 1). Combining a primer and a real-time probe has been tested for PCR with high-fidelity polymerase for the purpose of detection of viral infection [44]. This feature facilitates the design and generation of shorter amplified PCR products compared to conventional real-time PCR methods that require two primers and a separate probe. The resulting shorter PCR amplicons offer several distinct advantages. One such advantage pertains to ctDNA, which exists in patient plasma as short double-stranded DNA fragments with an average size of 166 bp, corresponding to the size of DNA wrapped around a nucleosome [45]. Conventional real-time PCR methods may fail to detect variants located at either end of these ctDNA fragments. However, PROMER technology can detect its target regardless of the variant’s position within the short ctDNA. In addition, PROMER technology can be effectively employed for real-time PCR of both AT-rich and GC-rich templates. For AT-rich targets, the optimization of probe binding conditions poses a challenge, as the melting temperature (TM) should exceed that of the primers by at least 5°C. This limitation is circumvented in PROMER PCR, as the PROMER functions as both a probe and a primer. In the case of GC-rich templates, the introduction of additional mismatch base pairs 3′ to the cleavage group (Fig. 1A) can resolve issues arising from high TMs. Another significant advantage is that PROMER PCR can also detect short targets such as miRNA. Furthermore, the high detection specificity of PROMER technology enables precise differentiation of a miRNA from its isotypes, which often exhibit subtle sequence and structural variations. A potential concern when combining a primer and a probe for real-time PCR is the increased likelihood of non-specific amplification. This typically occurs through the formation of primer dimers or non-specific annealing of the PROMER. However, our PROMER technology is designed to address this issue. The cleavage of primer dimers by RNase H2 denatures the primer dimers when they form. Furthermore, through the careful optimization of the PROMER sequence and PCR conditions, as described earlier, we can significantly reduce the chances of non-specific annealing of the PROMER. We believe these measures effectively address the potential concerns related to non-specific amplification, thereby enhancing the reliability and accuracy of our PROMER technology. Previously, the 3TEC-PCR [46] adopted a similar approach, utilizing *Tth* endonuclease IV in place of RNase H2 to facilitate sequence-specific cleavage of its primer-probe hybrid. While 3TEC-PCR demonstrated impressive sensitivity, its capacity to detect lower copy numbers of its target amidst a significant quantity of its wild-type counterpart is yet to be investigated.

We selected KRAS mutations as our testing templates for the PROMER technology because they represent a challenging and clinically relevant scenario. KRAS mutations are involved in several cancers such as colorectal, pancreatic, non-small cell lung cancers, oesophageal adenocarcinoma/gastroesophageal junction cancer, invasive ductal carcinoma, stomach adenocarcinoma, and undifferentiated endometrial carcinoma [42]. Moreover, KRAS mutations can occur at multiple positions and types within the same gene, requiring high specificity of detection [47]. Furthermore, KRAS mutations can vary among different tumor sites or liquid biopsies from the same patient due to tumor heterogeneity [48]. These clinical situations demand a simple, multiplexed, reliable detection technology for rare nucleic acids from the liquid biopsy. PROMER technology has the potential to provide a solution for comprehensive KRAS variant detection. Our multiplexed testing results and *in vivo* examination of ctDNA from animal models of human KRAS-driven cancers support the capability of PROMER technology (Table 5 and Fig. 2).

In summary, we have developed a new real-time PCR method, the PROMER technology that can precisely detect and quantify rare nucleic acids from liquid biopsies. The PROMER technology has several advantages over conventional real-time PCR methods, such as shorter amplicons, higher specificity, and multiplexing capability. We have demonstrated the applicability of the PROMER technology for the detection and quantification of KRAS mutations, which are clinically relevant for many cancers. The PROMER technology can provide valuable information for disease management, such as treatment selection, monitoring, prognosis, and resistance. We believe that the PROMER technology is a promising tool for precision medicine in various diseases.

## Supplementary Material

bpae041_Supplementary_Data

## Data Availability

The data underlying this article are available in the article and in its Online Supplementary material.
