# Supplementary material for: PROMER technology: A new real-time PCR tool enabling multiplex detection of point mutation with high specificity and sensitivity
Source: Biol Methods Protoc. 2024 Jun 4;9(1):bpae041. doi: 10.1093/biomethods/bpae041 (PMC11208725; doi:10.1093/biomethods/bpae041)
Supplement: bpae041_Supplementary_Data [file bpae041_supplementary_data.pdf]

PROMER technology: a new real-time PCR tool enabling multiplex detection of point mutation with high specificity and sensitivity.

AUTHORS

Hwanhee Nam[1], Esder Lee[2], Hichang Yang[2], Kyeyoon Lee[2], Taeho Kwak[2], Dain Kim[2], Hyemin Kim[2], Mihwa Yang[2], Younjoo Yang[2], Seungwan Son[2], Young-Hyean Nam[2], and Il Minn[1,3,*]

[1] Institute for NanoBioTechnology, Johns Hopkins University, Baltimore, Maryland, 21218, USA
[2] NuriBio Co., Ltd., F105, 66, Beolmal-ro, Dongan-gu, Anyang-si, Gyeonggi-Do, 14058 Republic of Korea
[3] Russell H. Morgan Department of Radiology and Radiological Science, Johns Hopkins Medical Institutions, Baltimore, Maryland, 21287, USA

* To whom correspondence should be addressed. Tel: +1-410-502-4024; Fax: +1-410-614-7822; Email: iminn1@jhmi.edu

SUPPLEMENTARY DATA

Supplementary Table S1. Template DNA and their sequences for the KRAS mutant and wildtype (WT) alleles. Mutated bases are highlighted in red.

Name	Mutation	Sequence
G12C	34G→T	5'-GAC TGA ATA TAA ACT TGT GGT AGT TGG AGC TTG TGG CGT AGG CAA GAG TGC CTT GAC GAT ACA GCT AAT TCA GAA TCA TTT TGT GGA CGA ATA TG-3'
G12S	34G→A	5'-GAC TGA ATA TAA ACT TGT GGT AGT TGG AGC TAG TGG CGT AGG CAA GAG TGC CTT GAC GAT ACA GCT AAT TCA GAA TCA TTT TGT GGA CGA ATA TG-3'
G12R	34G→C	5'-GAC TGA ATA TAA ACT TGT GGT AGT TGG AGC TCG TGG CGT AGG CAA GAG TGC CTT GAC GAT ACA GCT AAT TCA GAA TCA TTT TGT GGA CGA ATA TG-3'
G12V	35G→T	5'-GAC TGA ATA TAA ACT TGT GGT AGT TGG AGC TGT TGG CGT AGG CAA GAG TGC CTT GAC GAT ACA GCT AAT TCA GAA TCA TTT TGT GGA CGA ATA TG-3'
G12D	35G→A	5'-GAC TGA ATA TAA ACT TGT GGT AGT TGG AGC TGA TGG CGT AGG CAA GAG TGC CTT GAC GAT ACA GCT AAT TCA GAA TCA TTT TGT GGA CGA ATA TG-3'
G12A	35G→C	5'-GAC TGA ATA TAA ACT TGT GGT AGT TGG AGC TGC TGG CGT AGG CAA GAG TGC CTT GAC GAT ACA GCT AAT TCA GAA TCA TTT TGT GGA CGA ATA TG-3'
G13C	37G→T	5'-GAC TGA ATA TAA ACT TGT GGT AGT TGG AGC TGG TTG CGT AGG CAA GAG TGC CTT GAC GAT ACA GCT AAT TCA GAA TCA TTT TGT GGA CGA ATA TG-3'
G13S	37G→A	5'-GAC TGA ATA TAA ACT TGT GGT AGT TGG AGC TGG TAG CGT AGG CAA GAG TGC CTT GAC GAT ACA GCT AAT TCA GAA TCA TTT TGT GGA CGA ATA TG-3'
G13R	37G→C	5'-GAC TGA ATA TAA ACT TGT GGT AGT TGG AGC TGG TCG CGT AGG CAA GAG TGC CTT GAC GAT ACA GCT AAT TCA GAA TCA TTT TGT GGA CGA ATA TG-3'
G13V	38G→T	5'-GAC TGA ATA TAA ACT TGT GGT AGT TGG AGC TGG TGT CGT AGG CAA GAG TGC CTT GAC GAT ACA GCT AAT TCA GAA TCA TTT TGT GGA CGA ATA TG-3'
G13D	38G→A	5'-GAC TGA ATA TAA ACT TGT GGT AGT TGG AGC TGG TGA CGT AGG CAA GAG TGC CTT GAC GAT ACA GCT AAT TCA GAA TCA TTT TGT GGA CGA ATA TG-3'
G13A	38G→C	5'-GAC TGA ATA TAA ACT TGT GGT AGT TGG AGC TGG TGC CGT AGG CAA GAG TGC CTT GAC GAT ACA GCT AAT TCA GAA TCA TTT TGT GGA CGA ATA TG-3'
Q61H	183A→C	5'-ACC TGT CTC TTG GAT ATT CTC GAC ACA GCA GGT CAC GAG GAGTAC AGT GCA ATG AGG GAC CAG TAC ATG AGG ACT GGG GAG GGC TTT CTT TGT-3'
WT1	N/A	5'-GAC TGA ATA TAA ACT TGT GGT AGT TGG AGC TGG TGG CGT AGG CAA GAG TGC CTT GAC GAT ACA GCT AAT TCA GAA TCA TTT TGT GGA CGA ATA TG-3'
WT2	N/A	5'-ACC TGT CTC TTG GAT ATT CTC GAC ACA GCA GGT CAA GAG GAGTAC AGT GCA ATG AGG GAC CAG TAC ATG AGG ACT GGG GAG GGC TTT CTT TGT-3'

Supplementary Table S2. Sequences of the PROMERs and Primers.

	Name	Sequence (5′ → 3′)
PROMERs	G12C	**Cy5**/AAC TTG TGG TAG TTG GAG CTrU GT/**Q**
	G12S	**FAM**/AAC TTG TGG TAG TTG GAG CTrA GT/**Q**
	G12R	**HEX**/TAA ACT TGT GGT AGT TGG TGC TrCG TG/**Q**
	G12V	**HEX**/CTT GTG GTA GTT GGA GCT GrUT G/**Q**
	G12D	**FAM**/CTT GTG GTA GTT GGA GCT GrAT G/**Q**
	G12A	**Cy5**/CTT GTG GTA GTT GGT GCT GrCT G/**Q**
	G13C	**Cy5**/TGG TAG TTG GAG CTG GTrU GC/**Q**
	G13S	**FAM**/TGT GGT AGT TGG AGC TGG TrAG C/**Q**
	G13R	**HEX**/GGT AGT TGG AGC TGG TrCG C/**Q**
	G13D	**HEX**/GGT AGT TGG AGC TGG TGrA CG/**Q**
	G13A	**Cy5**/GGT AGT TGG AGC TGG TGrC CG/**Q**
	G13V	**FAM**/GGT AGT TGG AGC TGG TGrU CG/**Q**
	Q61H_C	**FAM**/CGA CAC AGC AGG TCAr CGA/**Q**
	Q61H_T	**FAM**/CTC GAC ACA GCA GGT CArU GA/**Q**
Primers	KRAS-rp (for G12/13)	CAT ATT CGT CCA CAA AAT GAT TCT G
	KRAS-rp (ForQ61)	CTC ATG TAC TGG TCC CTC ATT

Q: Iowa Black™ FQ Quancher.

Supplementary Table S3. List of the cell lines for KRAS mutants and wildtype.

KRAS mutant cell line						
No.	Mutation	Cell Lines (ATCC Cat#)	Zygosity	Gender	Tissue	Application
1	G12C	MIA PaCa-2 (CRL-1420)	Homozygous	Male	Pancreas	gDNA
2		NCI-H358 (CRL-5807)	Heterozygous	Male	Lung	cfDNA
3		SW1573 (CRL-2170)	Homozygous	Female	Lung	gDNA
4	G12S	A549 (CLL-185)	Homozygous	Male	Lung	gDNA/cfDNA
5	G12R	MDA-MB-134VI (HTB-23)	Heterozygous	Female	Breast	gDNA
6	G12V	SW620 (CCL-227)	Homozygous	Male	Colon	gDNA/cfDNA
7	G12D	SNU-C2B (CCL-250)	Homozygous	Female	Caecum	gDNA/cfDNA
8		Panc-1 (CRL-1469)	Heterozygous	Male	Pancreas	cfDNA
9	G12A	SW1116 (CCL-233)	Heterozygous	Male	Colon	gDNA/cfDNA
10		NCI-H2009 (CRL-5911)	Heterozygous	Female	Lung	gDNA/cfDNA
11	G13C	NCI-H1734 (CRL-5891)	Heterozygous	Female	Lung	gDNA
12	G13D	HCT-15 (CCL-225)	Heterozygous	Male	Colon	gDNA/cfDNA
13		MDA-MB-231 (HTB-26)	Heterozygous	Female	Breast	cfDNA
14	Q61H	NCI-H460 (HTB-177)	Homozygous	Male	Lung	gDNA/cfDNA
15	Wildtype	NCI-H1975 (CRL-5908)	Homozygous	Female	Lung	gDNA
15	Wildtype	HEK-293 (CRL-1573)	Homozygous	Female	Kidney	gDNA

Supplementary Table S4. PCR reaction setup and thermal-cycling conditions.

Thermal cycler setting

Thermal Cycler	Fluorophore	Threshold	Baseline
QunatSudio 3 and 5	Cy5, FAM, HEX	20,000	Auto-calculated
QunatSudio 12K Flex	Cy5	250,000	Auto-calculated
	FAM	250,000	Auto-calculated
	HEX	10,000	Auto-calculated
CFX96	Cy5	600	Auto-calculated
	FAM	600	Auto-calculated
	HEX	400	Auto-calculated

PROMER PCR with Synthetic Template

Component	Volume per reaction (µL)
5x Apta Taq DNA Master Mix	4
PROMER (10 µM)	0.5
Reverse Primer (10 µM)	0.5
RNase H2 (0.4 U/µL)	1
Synthetic Template	2
Nuclease Free Water	12

Cycling Step	Temperature (ºC)	Time (second)	Number of Cycle
Initial Denaturation	95	600	1
Denaturation	95	15	50
Annealing & Extension	64	60	

PROMER PCR with Genomic DNA and cfDNA

Component	Volume per reaction (µL)		
	G12C/R	G12V/A, G13C	G12D/S, G13D, Q61H
5x Apta Taq DNA Master Mix	4	4	2.8
AptaTaq Fast Buffer 5X	0	0	1.2
PROMER (10 µM)	0.5	0.5	0.5
Reverse Primer (10 µM)	0.5	0.5	0.5
RNase H2 (1 U/µL)	0.5	0.5	0.2
gDNA Template	4	4	4
$MgCl_2$ (25mM)	0.232	0	0

Nuclease Free Water	Up to 20 µL	Up to 20 µL	Up to 20 µL

Cycling Step	Temperature (ºC)	Time (second)	Number of Cycle
Initial Denaturation	95	600	1
Denaturation	95	10	40
Annealing & Extension	64	60	

Multiplexed PROMER PCR

Component	Volume per reaction (µL)	
	G12C/S/R	G12V/D/A
5x Apta Taq DNA Master Mix	4	4
RNase H2 (1 U/µL)	0.2	0
Template	4	4
Reverse Primer (10 µM)	0.5	0.5
G12C Promer(10uM)	0.9	0
G12S Promer(10uM)	0.165	0
G12R Promer(10uM)	0.45	0
G12V Promer(10uM)	0	0.4
G12D Promer(10uM)	0	0.2
G12A Promer(10uM)	0	0.4
Nuclease Free Water	Up to 20 µL	Up to 20 µL

Cycling Step	Temperature (ºC)	Time (second)	Number of Cycle
Initial Denaturation	95	600	1
Denaturation	95	10	40
Annealing & Extension	64	60	

TaqMan PCR with cfDNA

Component	Volume per reaction (µL)
2x TaqMan™ Gene Expression Master Mix	5
10x Custom TaqMan Gene Expression Assays	1
cfDNA Template	2
Nuclease Free Water	2

Cycling Step	Temperature (ºC)	Time (second)	Number of Cycle
Initial Denaturation	95	600	1
Pre-Denaturation	92	15	5
Pre-Cycle	58	60	
Denaturation	92	15	40

Annealing & Extension	60	60	

Supplementary Table S5. PROMER PCR Results with genomic DNAs of tested cell lines. Efficiency represents detectability analyzed by the Profit analysis. NTC: No template control

MIA PaCa-2 (G12C).

Copy Number	Ct Value	Ct Mean ± SD	CV	Efficiency
1×10^4	24.74	24.69 ± 0.11	0.43	1.02
	24.58			
	24.62			
	24.81			
5×10^3	25.58	25.3 ± 0.19	0.75	
	25.27			
	25.15			
	25.21			
1×10^3	27.84	27.86 ± 0.08	0.28	
	27.85			
	27.96			
	27.78			
5×10^2	28.55	28.55 ± 0.11	0.39	
	28.48			
	28.71			
	28.48			
1×10^2	31.37	31.31 ± 0.16	0.51	
	31.26			
	31.49			
	31.12			
1×10^1	34.93	34.68 ± 0.42	1.20	
	35.03			
	34.10			
	34.66			
1×10^0	37.56	37.4 ± 0.58	1.55	
	37.09			
	38.13			
	36.80			
NTC	ND	ND	ND	
	ND			
	ND			
	ND			

MDA-MB-134-VI (G12R)

Copy Number	Ct Value	Ct Mean ± SD	CV	Efficiency
1×10^4	27.30	27.37 ± 0.07	0.25	1.06
	27.38			
	27.46			
	27.34			
5×10^3	28.13	28.01 ± 0.19	0.69	
	28.21			
	27.92			
	27.78			

1×10^3	30.63	30.59 ± 0.11	0.37	
	30.44			
	30.72			
	30.58			
1×10^2	33.78	33.49 ± 0.33	0.97	
	33.75			
	33.26			
	33.16			
1×10^1	37.23	37.72 ± 0.71	1.87	
	38.62			
	37.10			
	37.94			
1×10^0	39.40	39.51 ± 0.36	0.91	
	39.22			
	ND			
	39.91			
NTC	ND	ND	ND	
	ND			
	ND			
	ND			

SW620 (G12V)

Copy Number	Ct Value	Ct Mean ± SD	CV	Efficiency
1×10^4	24.21	24.27 ± 0.134	0.55	0.93
	24.25			
	24.45			
	24.14			
5×10^3	25.21	25.18 ± 0.152	0.60	
	25.23			
	25.31			
	24.96			
1×10^3	27.77	27.78 ± 0.13	0.47	
	27.62			
	27.93			
	27.82			
1×10^2	31.19	31.09 ± 0.13	0.43	
	31.20			
	31.02			
	30.93			
1×10^1	34.37	34.55 ± 0.14	0.40	
	34.55			
	34.61			
	34.69			
1×10^0	37.66	38.38 ± 1.79	4.67	
	40.30			
	36.24			
	39.31			
NTC	ND	ND	ND	

	ND			
	ND			
	ND			

SW1116 (G12A)

Copy Number	Ct Value	Ct Mean ± SD	CV	Efficiency
1×10^4	23.95	23.92 ± 0.06	0.26	0.93
	23.87			
	23.86			
	23.99			
5×10^3	24.50	24.42 ± 0.15	0.60	
	24.59			
	24.31			
	24.29			
1×10^3	27.13	27.08 ± 0.07	0.27	
	27.03			
	27.15			
	27.00			
1×10^2	30.74	30.44 ± 0.23	0.75	
	30.47			
	30.20			
	30.36			
1×10^1	34.26	34.16 ± 0.19	0.56	
	34.28			
	33.88			
	34.25			
1×10^0	38.15	37.62 ± 0.60	1.61	
	36.96			
	37.74			
	ND			
NTC	ND	ND	ND	
	ND			
	ND			
	ND			

NCI-H1734 (G13C)

Copy Number	Ct Value	Ct Mean ± SD	CV	Efficiency
1×10^4	21.85	21.78 ± 0.10	0.45	0.97
	21.64			
	21.79			
	21.84			
5×10^3	22.25	22.24 ± 0.02	0.08	
	22.25			
	22.23			
	22.22			
1×10^3	24.63	24.56 ± 0.05	0.22	
	24.50			
	24.55			
	24.56			

1 × 10^2	28.05	27.86 ± 0.16	0.59	
	27.95			
	27.78			
	27.68			
1 × 10^1	31.02	31.27 ± 0.22	0.70	
	31.55			
	31.28			
	31.22			
1 × 10^0	36.98	35.25 ± 1.28	3.64	
	35.44			
	34.18			
	34.38			
NTC	ND	ND	ND	
	ND			
	ND			
	ND			

SNU-C2B (G12D)

Copy Number	Ct Value	Ct Mean ± SD	CV	Efficiency
1 × 10^4	25.50	25.31 ± 0.13	0.52	1.29
	25.21			
	25.25			
	25.28			
5 × 10^3	28.40	28.27 ± 0.10	0.35	
	28.23			
	28.17			
	28.28			
1 × 10^3	32.22	31.83 ± 0.27	0.86	
	31.67			
	31.61			
	31.83			
1 × 10^2	35.20	35.38 ± 0.90	2.55	
	34.63			
	36.69			
	35.01			
1 × 10^1	37.86	36.65 ± 0.87	2.36	
	36.57			
	35.84			
	36.31			
1 × 10^0	ND	36.68 ± 0.27	0.74	
	36.88			
	36.489			
	ND			
NTC	ND	ND	ND	
	ND			
	ND			
	ND			

A549 (G12S)

Copy Number	Ct Value	Ct Mean ± SD	CV	Efficiency
1×10^4	24.39	24.44 ± 0.14	0.59	1.04
	24.32			
	24.41			
	24.65			
1×10^3	27.68	27.74 ± 0.14	0.52	
	27.67			
	27.68			
	27.96			
1×10^2	30.86	30.86 ± 0.23	0.73	
	30.55			
	30.97			
	31.07			
1×10^1	34.57	34.26 ± 0.26	0.77	
	34.31			
	33.94			
	34.21			
1×10^0	38.45	37.29 ± 1.03	2.75	
	36.52			
	ND			
	36.90			
NTC	ND	ND	ND	
	ND			
	ND			
	ND			

HCT-15 (G13D)

Copy Number	Ct Value	Ct Mean ± SD	CV	Efficiency
1×10^4	25.71	25.65 ± 0.09	0.33	1.08
	25.63			
	25.53			
	25.71			
1×10^3	28.25	28.23 ± 0.06	0.21	
	28.26			
	28.14			
	28.25			
1×10^2	31.47	31.37 ± 0.11	0.35	
	31.36			
	31.43			
	31.22			
1×10^1	35.58	35.20 ± 0.30	0.86	
	34.95			
	35.31			
	34.98			
1×10^0	ND	37.90 ± 0.05	0.13	
	37.89			
	37.86			
	37.96			
NTC	ND	ND	ND	

	ND			
	ND			
	ND			

NCI-H460 (Q61H)

Copy Number	Ct Value	Ct Mean ± SD	CV	Efficiency
1×10^4	24.36	25.31 ± 0.15	0.57	1.04
	24.09			
	24.17			
	24.03			
5×10^3	27.35	28.27 ± 0.11	0.40	
	27.10			
	27.18			
	27.28			
1×10^3	31.15	31.83 ± 0.43	1.36	
	30.79			
	30.80			
	30.76			
1×10^2	33.80	35.38 ± 0.43	1.20	
	34.07			
	34.16			
	34.81			
1×10^1	36.64	36.65 ± 0.81	2.22	
	36.92			
	37.23			
	35.38			
1×10^0	ND	36.68 ± 0.27	0.74	
	37.64			
	37.67			
	37.19			
NTC	ND	ND	ND	
	ND			
	ND			
	ND			

Supplementary Table S6. PROMER and TaqMan PCR Results with genomic DNA from tested cell lines in the presence of KRAS wildtype DNA. NTC: No template control

MIA PaCa-2 (G12C)

	PROMER		TaqMan	
Copy Number of Mutant DNA	Ct Value	Ct Mean ± SD	Ct Value	Ct Mean ± SD
1×10^3	27.74	27.93 ± 0.18	29.08	29.07± 0.01
	27.95		29.07	
	28.09		NT	
1×10^2	31.64	31.63 ± 0.34	33.00	33.00 ± 0.00
	31.97		33.00	
	31.29		NT	
1×10^1	35.26	35.49 ± 0.21	36.82	37.48 ± 0.92
	35.58		38.13	
	35.65		NT	
1×10^0	37.31	37.59 ± 0.49	37.78	37.78 ± 0.00
	37.3		ND	
	38.15		NT	
0	ND	ND	ND	ND
	ND		ND	
	ND		ND	
NTC	ND	ND	ND	ND
	ND		ND	
	ND		ND	

MDA-MB-134-VI (G12R)

	PROMER		TaqMan	
Copy Number of Mutant DNA	Ct Value	Ct Mean ± SD	Ct Value	Ct Mean ± SD
1×10^3	30.28	30.31 ± 0.10	NT	NT
	30.23		NT	
	30.42		NT	
1×10^2	33.48	33.45 ± 0.31	NT	NT
	33.75		NT	
	33.13		NT	
1×10^1	37.05	37.03 ± 0.45	NT	NT
	36.57		NT	
	37.46		NT	
1×10^0	38.03	38.70 ± 0.94	NT	NT
	ND		NT	
	39.36		NT	
0	ND	ND	NT	NT
	ND		NT	
	ND		NT	
NTC	ND	ND	NT	NT
	ND		NT	
	ND		NT	

SW620 (G12V)

	PROMER		TaqMan	
Copy Number of Mutant DNA	Ct Value	Ct Mean ± SD	Ct Value	Ct Mean ± SD
1×10^3	27.75	27.75 ± 0.05	28.03	28.04 ± 0.05
	27.70		28.10	
	27.79		28.00	
1×10^2	31.09	30.89 ± 0.18	31.45	31.47 ± 0.04
	30.85		31.52	
	30.74		31.44	
1×10^1	34.40	34.62 ± 0.43	36.17	36.10 ± 0.08
	34.35		36.01	
	35.12		36.12	
1×10^0	39.38	38.54 ± 1.02	ND	40.49 ± 0.00
	37.41		40.49	
	38.83		ND	
0	ND	ND	ND	ND
	ND		ND	
	ND		ND	
NTC	ND	ND	ND	ND
	ND		ND	
	ND		ND	

SW1116 (G12A) for PROMER and NCI-H2009 (G12A) for TaqMan

	PROMER		TaqMan	
Copy Number of Mutant DNA	Ct Value	Ct Mean ± SD	Ct Value	Ct Mean ± SD
1×10^3	29.28	29.48 ± 0.17	29.53	29.56 ± 0.10
	29.57		29.48	
	29.59		29.66	
1×10^2	33.07	32.86 ± 0.21	33.14	33.07 ± 0.07
	32.65		33.03	
	32.87		33.03	
1×10^1	37.42	36.94 ± 0.43	38.26	37.74 ± 0.47
	36.81		37.64	
	36.60		37.32	
1×10^0	41.68	40.77 ± 1.28	38.67	39.02 ± 0.49
	39.87		ND	
	ND		39.36	
0	ND	ND	ND	ND
	ND		0 ND	
	ND		ND	
NTC	ND	ND	ND	ND
	ND		ND	
	ND		ND	

NCI-H1734 (G13C)

	PROMER		TaqMan	

Copy Number of Mutant DNA	Ct Value	Ct Mean ± SD	Ct Value	Ct Mean ± SD
1×10^3	26.80	26.89 ± 0.08	28.13	28.14 ± 0.04
	26.94		28.18	
	26.92		28.11	
1×10^2	30.38	30.12 ± 0.25	31.59	31.59 ± 0.02
	29.88		31.60	
	30.10		31.56	
1×10^1	34.16	34.45 ± 0.58	36.12	35.79 ± 0.37
	34.07		35.86	
	35.12		35.39	
1×10^0	38.09	38.04 ± 0.10	40.53	39.98 ± 0.75
	37.92		40.27	
	38.10		39.13	
0	ND	ND	ND	ND
	ND		ND	
	ND		ND	
NTC	ND	ND	ND	ND
	ND		ND	
	ND		ND	

SNU-C2B (G12D)

	PROMER		TaqMan	
Copy Number of Mutant DNA	Ct Value	Ct Mean ± SD	Ct Value	Ct Mean ± SD
1×10^3	29.18	28.77 ± 0.41	30.71	30.71 ± 0.02
	29.07		30.69	
	28.38		30.72	
	28.46		NT	
1×10^2	31.65	31.91 ± 0.23	34.15	34.11 ± 0.12
	31.92		34.20	
	31.87		33.98	
	32.20		NT	
1×10^1	35.10	35.64 ± 0.38	38.37	38.48 ± 0.10
	36.00		38.54	
	35.68		38.53	
	35.79		NT	
1×10^0	37.01	36.42 ± 0.46	40.26	40.59 ± 0.47
	35.98		ND	
	36.20		40.59	
	36.14		NT	
0	38.11	38.42 ± 0.55	ND	39.35 ± 0.00
	39.06		ND	
	38.09		39.35	
	ND		NT	
NTC	NT	NT	ND	ND
	NT		ND	
	NT		ND	

A549 (G12S)

	PROMER		TaqMan	
Copy Number of Mutant DNA	Ct Value	Ct Mean ± SD	Ct Value	Ct Mean ± SD
1×10^3	27.81	27.74 ± 0.11	28.52	28.14 ± 0.39
	27.84		28.18	
	27.61		27.74	
	27.67		NT	
1×10^2	31.44	31.33 ± 0.17	32.14	31.85 ± 0.41
	31.08		32.03	
	31.38		31.38	
	31.43		NT	
1×10^1	34.53	34.66 ± 0.12	35.86	36.06 ± 0.99
	34.81		37.14	
	34.63		35.19	
	34.65		NT	
1×10^0	37.27	38.25 ± 1.08	ND	37.63 ± 0.89
	37.43		38.26	
	39.51		37.00	
	38.80		NT	
0	39.21	39.46 ± 0.35	ND	39.82 ± 0.80
	39.71		40.39	
	ND		39.26	
	ND		NT	
NTC	NT		ND	ND
	NT		ND	
	NT		ND	

HCT-15 (G13D)

	PROMER		TaqMan	
Copy Number of Mutant DNA	Ct Value	Ct Mean ± SD	Ct Value	Ct Mean ± SD
1×10^3	29.25	29.13 ± 0.14	32.24	32.17 ± 0.07
	29.04		32.10	
	29.25		32.17	
	28.97		NT	
1×10^2	32.53	32.44 ± 0.11	35.22	35.53 ± 0.27
	32.54		35.64	
	32.36		35.72	
	32.33		NT	
1×10^1	36.45	35.86 ± 0.53	40.45	40.24 ± 0.27
	36.05		40.33	
	35.75		39.93	
	35.20		NT	
1×10^0	38.48	38.14 ± 0.50	ND	ND
	37.55		ND	
	37.91		ND	
	38.61		NT	
0	38.99	39.19 ± 0.83	ND	ND
	38.15		ND	

	40.13		ND	
	39.48		NT	
NTC	NT		ND	ND
	NT		ND	
	NT		ND	

NCI-H460 (Q61H)

	PROMER		TaqMan	
Copy Number of Mutant DNA	Ct Value	Ct Mean ± SD	Ct Value	Ct Mean ± SD
1×10^3	27.89	28.77 ± 0.10	29.39	29.45 ± 0.10
	28.02		29.57	
	28.11		29.39	
	27.92		NT	
1×10^2	31.42	31.91 ± 0.18	32.83	32.89 ± 0.24
	31.57		32.68	
	31.22		33.15	
	31.61		NT	
1×10^1	34.93	35.64 ± 0.16	ND	38.09 ± 0.92
	35.12		37.43	
	35.12		38.74	
	35.34		NT	
1×10^0	38.62	36.33 ± 0.67	38.68	38.04 ± 0.61
	ND		37.46	
	ND		37.98	
	37.67		NT	
0	ND	ND	ND	ND
	ND		ND	
	ND		ND	
	ND		ND	
NTC	NT	NT	ND	ND
	NT		ND	
	NT		ND	

Supplementary Table S7. PROMER and TaqMan results and standard curve with genomic DNA from indicated cell line.

MIA-PaCa-2 (G12C)

Copy #	Log2	PROMER ct mean	TaqMan ct mean
562.50	9.135709	27.46	25.24
281.25	8.135709	28.43	26.20
140.63	7.135709	29.48	27.35
70.31	6.135709	30.42	28.39
35.16	5.135709	31.42	29.50
17.58	4.135709	32.60	30.94
8.79	3.135709	33.54	31.65
4.39	2.135709	35.68	34.00
2.20	1.135709	35.36	33.34
1.10	0.135709	36.26	ND

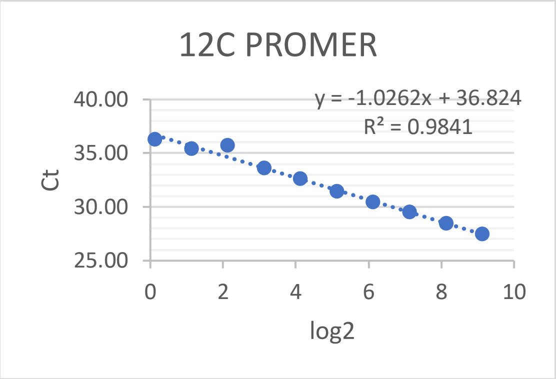

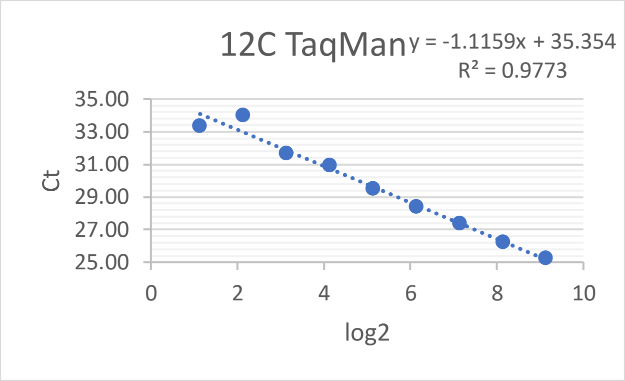

A549 (G12S)

Copy #	Log2	PROMER ct mean	TaqMan ct mean
562.50	9.135709	27.11	25.47
281.25	8.135709	28.13	26.52
140.63	7.135709	29.06	27.53
70.31	6.135709	30.06	28.86
35.16	5.135709	31.39	29.70
17.58	4.135709	31.76	30.43
8.79	3.135709	33.02	32.14
4.39	2.135709	34.23	32.31
2.20	1.135709	34.87	34.94
1.10	0.135709	36.32	34.39

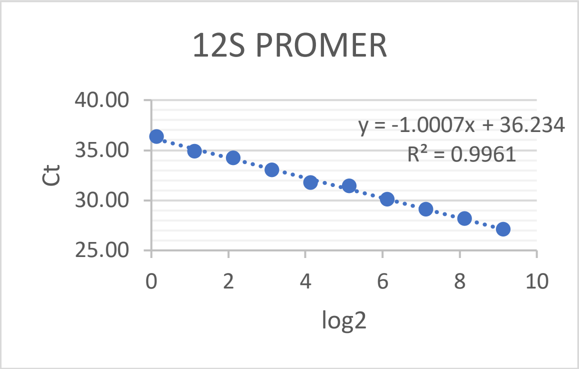

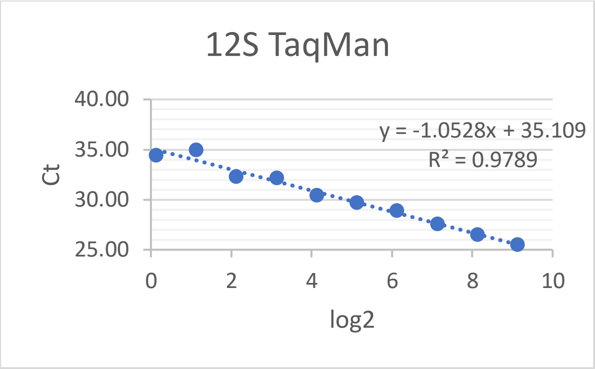

SW620 (G12V)

Copy #	Log2	PROMER Ct mean	TaqMan Ct mean
562.50	9.135709	27.75	23.96
281.25	8.135709	28.71	25.14
140.63	7.135709	29.59	26.24
70.31	6.135709	30.61	27.26
35.16	5.135709	31.65	28.81
17.58	4.135709	32.69	29.50
8.79	3.135709	34.13	30.67
4.39	2.135709	35.57	31.77
2.20	1.135709	35.87	33.65
1.10	0.135709	37.37	37.77

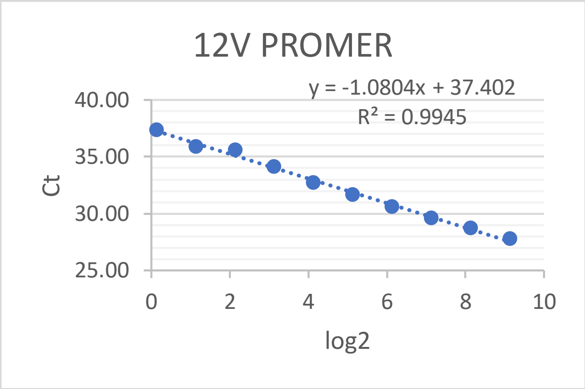

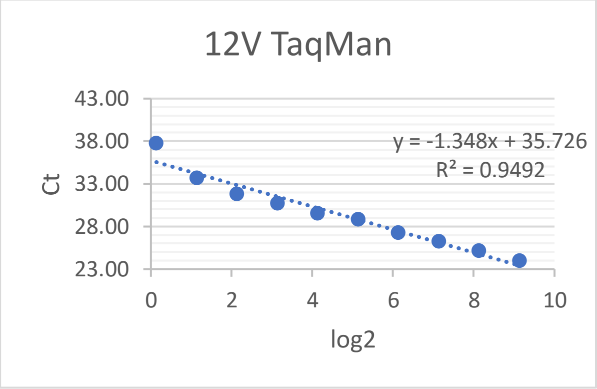

SNU-C2B (G12D)

Copy #	Log2	PROMER ct mean	TaqMan ct mean
562.50	9.135709	29.15	25.62
281.25	8.135709	30.07	27.05
140.63	7.135709	31.10	27.99
70.31	6.135709	32.03	29.11
35.16	5.135709	33.23	30.44
17.58	4.135709	34.24	31.30
8.79	3.135709	35.14	32.52
4.39	2.135709	36.01	33.57
2.20	1.135709	37.54	33.75

1.10	0.135709	37.76	35.24

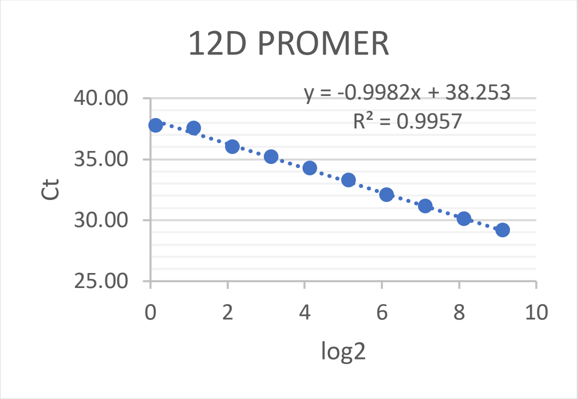

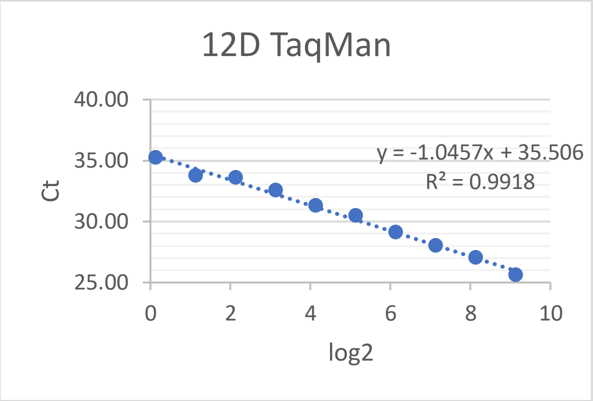

NCI-H2009 (G12A)

Copy #	Log2	PROMER ct mean	TaqMan ct mean
562.50	9.135709	29.75	25.56
281.25	8.135709	30.81	26.77
140.63	7.135709	31.93	27.97
70.31	6.135709	32.78	28.86
35.16	5.135709	33.77	30.03
17.58	4.135709	34.89	31.58
8.79	3.135709	35.72	32.04
4.39	2.135709	37.17	32.89
2.20	1.135709	37.68	35.72
1.10	0.135709	37.89	33.91

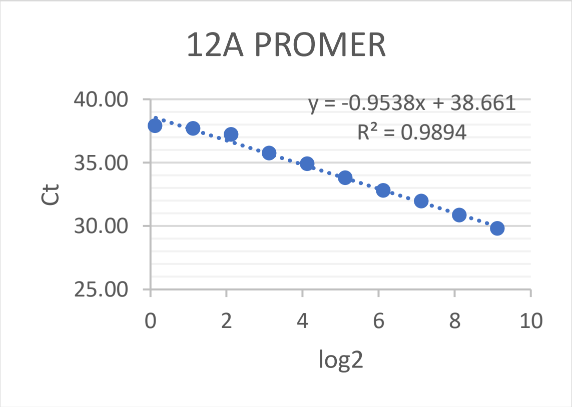

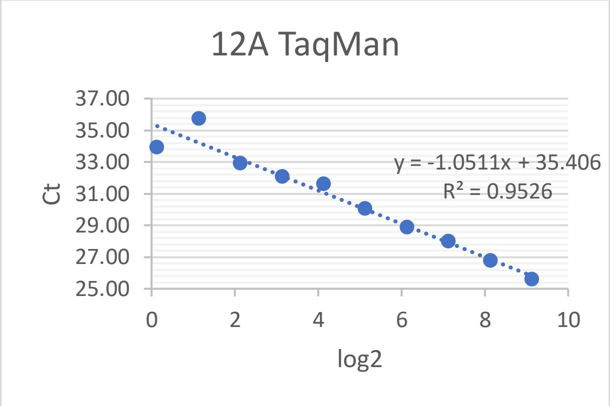

NCI-H1734 (G13C)

Copy #	Log2	PROMER ct mean	TaqMan ct mean
562.50	9.135709	26.74	23.93
281.25	8.135709	27.79	25.05
140.63	7.135709	28.66	26.24
70.31	6.135709	29.74	27.24

35.16	5.135709	30.87	28.48
17.58	4.135709	31.58	29.27
8.79	3.135709	32.85	30.45
4.39	2.135709	33.79	31.46
2.20	1.135709	34.95	33.15
1.10	0.135709	36.48	34.56

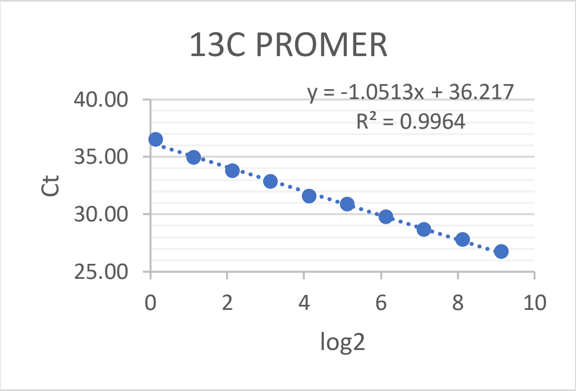

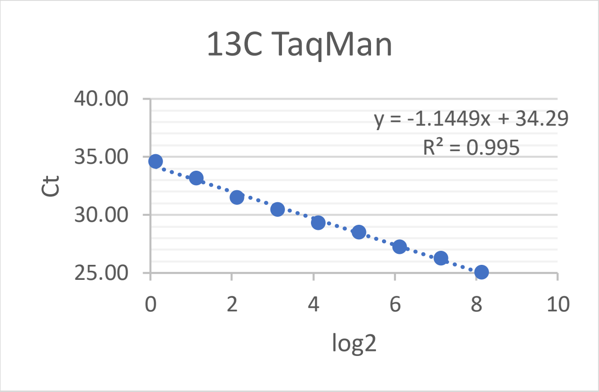

HCT-15 (G13D)

Copy #	Log2	PROMER ct mean	TaqMan ct mean
562.50	9.135709	30.04	26.68
281.25	8.135709	31.11	27.63
140.63	7.135709	31.99	28.65
70.31	6.135709	33.13	29.57
35.16	5.135709	34.60	31.40
17.58	4.135709	35.87	33.29
8.79	3.135709	37.96	34.02
4.39	2.135709	38.13	35.18
2.20	1.135709	39.02	38.26
1.10	0.135709	39.28	38.09

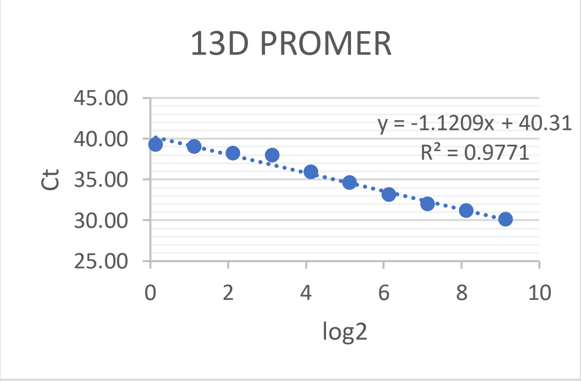

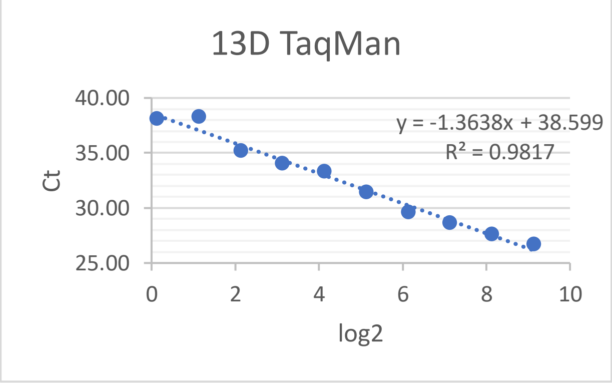

NCI-H460 (Q61H)

Copy #	Log2	PROMER ct mean	TaqMan ct mean

562.50	9.135709	29.47	24.29
281.25	8.135709	30.46	25.48
140.63	7.135709	31.65	26.85
70.31	6.135709	32.77	27.58
35.16	5.135709	33.96	28.19
17.58	4.135709	35.15	30.29
8.79	3.135709	35.52	32.72
4.39	2.135709	38.26	ND
2.20	1.135709	37.01	ND
1.10	0.135709	37.92	39.12

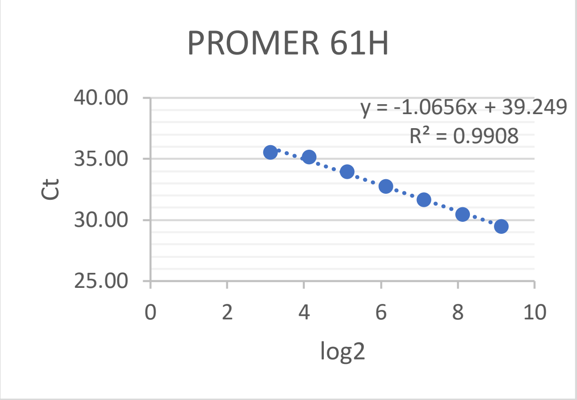

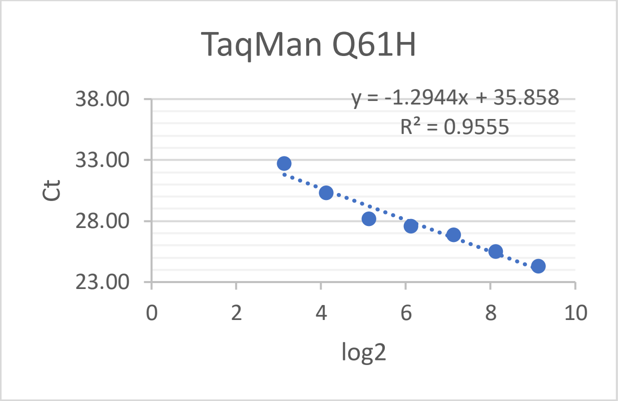

Supplementary Table S8. Detection Specificity of G12 variant (G12C/S/R/V/D/A) PROMERS.

<G12C>

PROMER	Sample		Ct									
G12C (Cy5)	NTC		NA	NA	NA	NA	NA	NA	NA	NA	NA	NA
			NA	NA	NA	NA	NA	NA	NA	NA	NA	NA
			NA	NA	NA	NA	NA	NA	NA	NA	NA	NA
			NA	NA	NA	NA	NA	NA	NA	NA	NA	NA
			NA	NA	NA	NA	NA	NA	NA	NA	NA	NA
			NA	NA	NA	NA	NA	NA	NA	NA	NA	NA
			NA	NA	NA	NA	NA	NA	NA	NA	NA	NA
			NA	NA	NA	NA	NA	NA	NA	NA	NA	NA
	WT (HEK293)	15ng	NA	NA	NA	NA	NA	NA	NA	NA	NA	NA
			NA	NA	NA	NA	NA	NA	NA	NA	NA	NA
		30ng	NA	NA	NA	NA	NA	NA	NA	NA	NA	NA
			NA	NA	NA	NA	NA	NA	NA	NA	NA	NA
		45ng	NA	NA	NA	NA	NA	NA	NA	NA	NA	NA
			NA	NA	NA	NA	NA	NA	NA	NA	NA	NA
		60ng	NA	NA	NA	NA	NA	NA	NA	NA	NA	NA
			NA	NA	NA	NA	NA	NA	NA	NA	NA	NA
	G12C (SW1573)	15ng	28.75	28.43	28.21	28.42	28.99	28.59	28.75	28.43	28.21	28.42
			28.52	28.22	28.32	28.47	29.07	28.61	28.52	28.22	28.32	28.47
		30ng	28.19	27.82	27.90	28.04	28.47	27.96	28.19	27.82	27.90	28.04
			28.28	27.82	27.74	27.99	28.35	27.69	28.28	27.82	27.74	27.99
		45ng	28.20	27.34	27.61	27.09	28.34	27.83	28.20	27.34	27.61	27.09
			28.00	27.32	27.44	27.22	28.35	27.70	28.00	27.32	27.44	27.22
		60ng	27.74	27.27	27.32	27.35	28.46	27.71	27.74	27.27	27.32	27.35
			27.33	27.06	26.88	26.98	28.13	27.55	27.33	27.06	26.88	26.98
	G12S (A549)	15ng	NA	NA	NA	NA	NA	NA	NA	NA	NA	NA
			NA	NA	NA	NA	NA	NA	NA	NA	NA	NA
		30ng	NA	NA	NA	NA	NA	NA	NA	NA	NA	NA
			NA	NA	NA	NA	NA	NA	NA	NA	NA	NA
		45ng	NA	NA	NA	NA	NA	NA	NA	NA	NA	NA
			NA	NA	NA	NA	NA	NA	NA	NA	NA	NA
		60ng	NA	NA	NA	NA	NA	NA	NA	NA	NA	NA
			NA	NA	NA	NA	NA	NA	NA	NA	NA	NA
	G12R (MDA-MB-134VI)	15ng	NA	NA	NA	NA	NA	NA	NA	NA	NA	NA
			NA	NA	NA	NA	NA	NA	NA	NA	NA	NA
		30ng	NA	NA	NA	NA	NA	NA	NA	NA	NA	NA
			NA	NA	NA	NA	NA	NA	NA	NA	NA	NA
		45ng	NA	NA	NA	NA	NA	NA	NA	NA	NA	NA
			NA	NA	NA	NA	NA	NA	NA	NA	NA	NA
		60ng	NA	NA	NA	NA	NA	NA	NA	NA	NA	NA
			NA	NA	NA	NA	NA	NA	NA	NA	NA	NA
		15ng	NA	NA	NA	NA	NA	NA	NA	NA	NA	NA
			NA	NA	NA	NA	NA	NA	NA	NA	NA	NA

	G12V (SW620)	30ng	NA	NA	NA	NA	NA	NA	NA	NA	NA	NA
			NA	NA	NA	NA	NA	NA	NA	NA	NA	NA
		45ng	NA	NA	NA	NA	NA	NA	NA	NA	NA	NA
			NA	NA	NA	NA	NA	NA	NA	NA	NA	NA
		60ng	NA	NA	NA	NA	NA	NA	NA	NA	NA	NA
			NA	NA	NA	NA	NA	NA	NA	NA	NA	NA
	G12A (NCI-H2009)	15ng	NA	NA	NA	NA	NA	NA	NA	NA	NA	NA
			NA	NA	NA	NA	NA	NA	NA	NA	NA	NA
		30ng	NA	NA	NA	NA	NA	NA	NA	NA	NA	NA
			NA	NA	NA	NA	NA	NA	NA	NA	NA	NA
		45ng	NA	NA	NA	NA	NA	NA	NA	NA	NA	NA
			NA	NA	NA	NA	NA	NA	NA	NA	NA	NA
		60ng	NA	NA	NA	NA	NA	NA	NA	NA	NA	NA
			NA	NA	NA	NA	NA	NA	NA	NA	NA	NA
	G12D SNU-C2B	15ng	NA	NA	NA	NA	NA	NA	NA	NA	NA	NA
			NA	NA	NA	NA	NA	NA	NA	NA	NA	NA
		30ng	NA	NA	NA	NA	NA	NA	NA	NA	NA	NA
			NA	NA	NA	NA	NA	NA	NA	NA	NA	NA
		45ng	NA	NA	NA	NA	NA	NA	NA	NA	NA	NA
			NA	NA	NA	NA	NA	NA	NA	NA	NA	NA
		60ng	NA	NA	NA	NA	NA	NA	NA	NA	NA	NA
			NA	NA	NA	NA	NA	NA	NA	NA	NA	NA

NA : Not amplified; NTC : No template control

<G12S>

PROMER	Sample		Ct									
G12S (FAM)	NTC		NA	NA	NA	NA	NA	NA	NA	NA	NA	NA
			NA	NA	NA	NA	NA	NA	NA	NA	NA	NA
			NA	NA	NA	NA	NA	NA	NA	NA	NA	NA
			NA	NA	NA	NA	NA	NA	NA	NA	NA	NA
			NA	NA	NA	NA	NA	NA	NA	NA	NA	NA
			NA	NA	NA	NA	NA	NA	NA	NA	NA	NA
			NA	NA	NA	NA	NA	NA	NA	NA	NA	NA
			NA	NA	NA	NA	NA	NA	NA	NA	NA	NA
	WT (HEK293)	15ng	NA	NA	NA	NA	NA	NA	NA	NA	NA	NA
			NA	NA	NA	NA	NA	NA	NA	NA	NA	NA
		30ng	NA	NA	NA	NA	NA	NA	NA	NA	NA	NA
			NA	NA	NA	NA	NA	NA	39.49	NA	39.00	NA
		45ng	NA	NA	NA	NA	NA	38.82	NA	NA	NA	NA
			NA	NA	NA	NA	NA	39.63	39.83	39.81	39.44	NA
		60ng	NA	NA	NA	NA	NA	NA	NA	NA	NA	NA
			NA	NA	NA	NA	NA	NA	NA	NA	39.89	NA
	G12C (SW1573)	15ng	NA	NA	NA	NA	NA	NA	NA	NA	NA	NA
			NA	NA	NA	NA	NA	NA	NA	NA	NA	NA
		30ng	NA	NA	NA	NA	NA	NA	NA	NA	NA	NA
			NA	NA	NA	NA	NA	NA	NA	NA	NA	NA
		45ng	NA	NA	NA	NA	NA	NA	NA	NA	NA	NA
			NA	NA	NA	NA	NA	NA	NA	NA	NA	NA
		60ng	NA	NA	NA	NA	NA	NA	NA	NA	NA	NA
			NA	NA	NA	NA	NA	NA	NA	NA	NA	NA
	G12S (A549)	15ng	27.52	27.46	27.28	26.99	27.41	27.22	27.52	27.46	27.28	26.99
			28.04	27.92	27.77	27.27	28.11	27.75	28.04	27.92	27.77	27.27
		30ng	26.96	26.84	26.66	26.36	26.85	26.73	26.96	26.84	26.66	26.36
			26.46	26.61	26.30	26.17	26.70	26.28	26.46	26.61	26.30	26.17
		45ng	25.78	26.26	25.85	25.40	25.63	25.62	25.78	26.26	25.85	25.40
			26.06	26.67	26.12	25.66	26.36	26.00	26.06	26.67	26.12	25.66
		60ng	26.16	25.62	26.40	25.89	26.30	26.21	26.16	25.62	26.40	25.89
			25.96	25.65	26.05	25.77	25.92	26.03	25.96	25.65	26.05	25.77
	G12R (MDA-MB-134VI)	15ng	NA	NA	NA	NA	NA	NA	NA	NA	NA	NA
			NA	NA	NA	NA	NA	39.65	NA	NA	NA	NA
		30ng	NA	NA	NA	NA	NA	NA	NA	NA	NA	NA
			NA	NA	NA	NA	NA	NA	NA	NA	NA	NA
		45ng	NA	NA	NA	NA	NA	NA	NA	NA	NA	NA
			NA	NA	NA	NA	NA	NA	NA	NA	NA	NA
		60ng	NA	NA	NA	NA	NA	NA	NA	NA	NA	NA
			NA	NA	NA	NA	NA	NA	NA	39.91	NA	NA
	G12V (SW620)	15ng	NA	NA	NA	NA	NA	NA	NA	NA	NA	NA
			NA	NA	NA	NA	NA	NA	NA	NA	NA	NA
		30ng	NA	NA	NA	NA	NA	NA	NA	NA	NA	NA
			NA	NA	NA	NA	NA	NA	NA	NA	NA	NA
		45ng	NA	NA	NA	NA	NA	NA	NA	NA	NA	NA
			NA	NA	NA	NA	NA	NA	NA	NA	NA	NA

		60ng	NA	NA	NA	NA	NA	NA	NA	NA	NA	NA
			NA	NA	NA	NA	NA	NA	NA	NA	NA	NA
	G12A (NCI-H2009)	15ng	NA	NA	NA	NA	NA	NA	NA	NA	NA	NA
			NA	NA	NA	NA	NA	NA	NA	NA	NA	NA
		30ng	NA	NA	NA	NA	NA	NA	NA	NA	NA	NA
			NA	NA	NA	NA	NA	NA	NA	NA	NA	NA
		45ng	NA	NA	NA	NA	NA	NA	NA	NA	NA	NA
			NA	NA	NA	NA	NA	NA	NA	39.54	NA	NA
		60ng	NA	NA	NA	NA	NA	NA	NA	NA	NA	NA
			NA	NA	NA	NA	NA	NA	NA	NA	NA	NA
	G12D SNU-C2B	15ng	NA	NA	NA	NA	NA	NA	NA	NA	NA	NA
			NA	NA	NA	NA	NA	NA	NA	NA	NA	NA
		30ng	NA	NA	NA	NA	NA	NA	NA	NA	NA	NA
			NA	NA	NA	NA	NA	NA	NA	NA	NA	NA
		45ng	NA	NA	NA	NA	NA	NA	NA	NA	NA	NA
			NA	NA	NA	NA	NA	NA	NA	NA	NA	NA
		60ng	NA	NA	NA	NA	NA	NA	NA	NA	NA	NA
			NA	NA	NA	NA	NA	NA	NA	NA	NA	NA

NA : Not amplified; NTC : No template control

<G12R>

PROMER	Sample		Ct									
G12R (Hex)	NTC		NA	NA	NA	NA	NA	NA	NA	NA	NA	NA
			NA	NA	NA	NA	NA	NA	NA	NA	NA	NA
			NA	NA	NA	NA	NA	NA	NA	NA	NA	NA
			NA	NA	NA	NA	NA	NA	NA	NA	NA	NA
			NA	NA	NA	NA	NA	NA	NA	NA	NA	NA
			NA	NA	NA	NA	NA	NA	NA	NA	NA	NA
			NA	NA	NA	NA	NA	NA	NA	NA	NA	NA
			NA	NA	NA	NA	NA	NA	NA	NA	NA	NA
	WT (HEK293)	15ng	NA	NA	NA	NA	NA	NA	NA	NA	NA	NA
			NA	NA	NA	NA	NA	NA	NA	NA	NA	NA
		30ng	NA	NA	NA	NA	NA	NA	NA	NA	NA	NA
			NA	NA	NA	NA	NA	NA	NA	NA	NA	NA
		45ng	NA	NA	NA	NA	NA	NA	NA	NA	NA	NA
			NA	NA	NA	NA	NA	NA	NA	NA	NA	NA
		60ng	NA	NA	NA	NA	NA	NA	NA	NA	NA	NA
			NA	NA	NA	NA	NA	NA	NA	NA	NA	NA
	G12C (SW1573)	15ng	NA	NA	NA	NA	NA	NA	NA	NA	NA	NA
			NA	NA	NA	NA	NA	NA	NA	NA	NA	NA
		30ng	NA	NA	NA	NA	NA	NA	NA	NA	NA	NA
			NA	NA	NA	NA	NA	39.26	NA	39.53	39.65	NA
		45n	NA	NA	NA	NA	NA	NA	NA	NA	NA	NA
			NA	NA	NA	NA	NA	NA	39.74	39.85	NA	NA
		60ng	NA	NA	NA	NA	NA	NA	NA	NA	NA	NA
			NA	NA	NA	NA	NA	NA	NA	39.56	NA	NA
	G12S (A549)	15ng	NA	NA	NA	NA	NA	NA	NA	NA	NA	NA
			NA	NA	NA	NA	NA	NA	NA	NA	NA	NA
		30ng	NA	NA	NA	NA	NA	NA	NA	NA	NA	NA
			NA	NA	NA	NA	NA	NA	NA	NA	NA	NA
		45ng	NA	NA	NA	NA	NA	NA	NA	NA	NA	NA
			NA	NA	NA	NA	NA	NA	NA	NA	NA	NA
		60ng	NA	NA	NA	NA	NA	NA	NA	NA	NA	NA
			NA	NA	NA	NA	NA	NA	NA	NA	NA	NA
	G12R (MDA-MB-134VI)	15ng	28.84	29.70	29.38	29.34	30.49	28.59	28.84	29.70	29.38	29.34
			29.47	30.86	29.85	29.79	30.82	29.29	29.47	30.86	29.85	29.79
		30ng	28.52	29.99	29.07	29.07	29.79	28.34	28.52	29.99	29.07	29.07
			27.86	29.24	28.30	27.94	28.57	27.43	27.86	29.24	28.30	27.94
		45ng	27.50	28.53	28.06	27.34	28.00	27.00	27.50	28.53	28.06	27.34
			28.34	29.07	28.43	27.68	28.31	27.29	28.34	29.07	28.43	27.68
		60ng	28.84	29.41	29.19	28.33	28.80	27.79	28.84	29.41	29.19	28.33
			28.07	28.70	28.76	27.74	28.25	27.05	28.07	28.70	28.76	27.74
	G12V (SW620)	15ng	NA	NA	NA	NA	NA	NA	NA	NA	NA	NA
			NA	NA	NA	NA	NA	NA	NA	NA	NA	NA
		30ng	NA	NA	NA	NA	NA	NA	NA	NA	NA	NA
			NA	NA	NA	NA	NA	NA	NA	NA	NA	NA
		45ng	NA	NA	NA	NA	NA	NA	NA	NA	NA	NA
			NA	NA	NA	NA	NA	NA	NA	NA	NA	NA

		60ng	NA	NA	NA	NA	NA	NA	NA	NA	NA	NA
			NA	NA	NA	NA	NA	NA	NA	NA	NA	NA
	G12A (NCI-H2009)	15ng	NA	NA	NA	NA	NA	NA	NA	NA	NA	NA
			NA	NA	NA	NA	NA	NA	NA	NA	NA	NA
		30ng	NA	NA	NA	NA	NA	NA	NA	NA	NA	NA
			NA	NA	NA	NA	NA	NA	NA	NA	NA	NA
		45ng	NA	NA	NA	NA	NA	NA	NA	NA	NA	NA
			NA	NA	NA	NA	NA	NA	NA	NA	NA	NA
		60ng	NA	NA	NA	NA	NA	NA	NA	NA	NA	NA
			NA	NA	NA	NA	NA	NA	NA	NA	NA	NA
	G12D SNU-C2B	15ng	NA	NA	NA	NA	NA	NA	NA	NA	NA	NA
			NA	NA	NA	NA	NA	NA	NA	NA	NA	NA
		30ng	NA	NA	NA	NA	NA	NA	NA	NA	NA	NA
			NA	NA	NA	NA	NA	NA	NA	NA	NA	NA
		45ng	NA	NA	NA	NA	NA	NA	NA	NA	NA	NA
			NA	NA	NA	NA	NA	NA	NA	NA	NA	NA
		60ng	NA	NA	NA	NA	NA	NA	NA	NA	NA	NA
			NA	NA	NA	NA	NA	NA	NA	NA	NA	NA

NA : Not amplified; NTC : No template control

<G12V>

PROMER	Sample		Ct									
G12V (Hex)	NTC		NA	NA	NA	NA	NA	NA	NA	NA	NA	NA
			NA	NA	NA	NA	NA	NA	NA	NA	NA	NA
			NA	NA	NA	NA	NA	NA	NA	NA	NA	NA
			NA	NA	NA	NA	NA	NA	NA	NA	NA	NA
			NA	NA	NA	NA	NA	NA	NA	NA	NA	NA
			NA	NA	NA	NA	NA	NA	NA	NA	NA	NA
			NA	NA	NA	NA	NA	NA	NA	NA	NA	NA
			NA	NA	NA	NA	NA	NA	NA	NA	NA	NA
	WT (HEK293)	15ng	NA	NA	NA	NA	NA	NA	NA	NA	NA	NA
			NA	NA	NA	NA	NA	39.41	NA	NA	NA	NA
		30ng	NA	NA	NA	NA	NA	NA	NA	NA	NA	NA
			NA	NA	NA	NA	NA	NA	NA	NA	NA	NA
		45ng	NA	NA	NA	NA	NA	NA	NA	NA	NA	NA
			NA	NA	NA	NA	NA	NA	NA	NA	NA	NA
		60ng	NA	NA	NA	NA	NA	NA	NA	NA	NA	NA
			NA	NA	NA	NA	NA	NA	NA	NA	NA	NA
	G12C (SW1573)	15ng	NA	NA	NA	NA	NA	NA	NA	NA	NA	NA
			NA	NA	NA	NA	NA	NA	NA	NA	NA	NA
		30ng	NA	NA	NA	NA	NA	NA	NA	NA	NA	NA
			NA	NA	NA	NA	NA	NA	NA	NA	NA	NA
		45ng	NA	NA	NA	NA	NA	NA	NA	NA	NA	NA
			NA	NA	NA	NA	NA	NA	NA	NA	NA	NA
		60ng	NA	NA	NA	NA	NA	NA	NA	NA	NA	NA
			NA	NA	NA	NA	NA	NA	NA	NA	NA	NA
	G12S (A549)	15ng	NA	NA	NA	NA	NA	NA	NA	NA	NA	NA
			NA	NA	NA	NA	NA	NA	NA	NA	NA	NA
		30ng	NA	NA	NA	NA	NA	NA	NA	NA	NA	NA
			NA	NA	NA	NA	NA	NA	NA	NA	NA	NA
		45ng	NA	NA	NA	NA	NA	NA	NA	NA	NA	NA
			NA	NA	NA	NA	NA	NA	NA	NA	NA	NA
		60ng	NA	NA	NA	NA	NA	NA	NA	NA	NA	NA
			NA	NA	NA	NA	NA	NA	NA	NA	NA	NA
	G12R (MDA-MB-134VI)	15ng	NA	NA	NA	NA	NA	NA	NA	NA	NA	NA
			NA	NA	NA	NA	NA	NA	NA	NA	NA	NA
		30ng	NA	NA	NA	NA	NA	NA	NA	NA	NA	NA
			NA	NA	NA	NA	NA	NA	NA	NA	NA	NA
		45ng	NA	NA	NA	NA	NA	NA	NA	NA	NA	NA
			NA	NA	NA	NA	NA	NA	NA	NA	NA	NA
		60ng	NA	NA	NA	NA	NA	NA	NA	NA	NA	NA
			NA	NA	NA	NA	NA	NA	NA	NA	NA	NA
	G12V (SW620)	15ng	25.52	25.68	25.34	25.41	25.73	25.35	25.52	25.68	25.34	25.41
			25.43	25.73	25.42	25.38	25.82	25.33	25.43	25.73	25.42	25.38
		30ng	24.81	24.88	24.66	24.67	24.93	24.55	24.81	24.88	24.66	24.67
			24.57	24.73	24.59	24.56	24.74	24.57	24.57	24.73	24.59	24.56
		45ng	24.09	24.35	24.23	24.30	24.50	24.31	24.09	24.35	24.23	24.30
			24.21	24.34	24.17	24.32	24.51	24.33	24.21	24.34	24.17	24.32

		60ng	24.08	24.13	24.09	24.09	24.21	24.05	24.08	24.13	24.09	24.09
			23.91	23.92	23.88	23.99	24.12	23.94	23.91	23.92	23.88	23.99
	G12A (NCI-H2009)	15ng	NA	NA	NA	NA	NA	NA	NA	NA	NA	NA
			39.07	NA	NA	NA	NA	NA	NA	NA	NA	NA
		30ng	NA	NA	NA	NA	NA	NA	NA	NA	NA	NA
			NA	NA	39.62	NA	NA	NA	NA	NA	39.40	NA
		45ng	38.98	NA	NA	39.60	NA	NA	39.85	NA	NA	NA
			38.66	NA	NA	NA	39.43	39.24	NA	NA	NA	NA
		60ng	NA	NA	NA	NA	NA	NA	39.80	NA	NA	NA
			NA	39.37	NA	39.89	38.29	NA	39.63	NA	NA	NA
	G12D SNU-C2B	15ng	NA	NA	NA	NA	NA	NA	NA	NA	NA	NA
			NA	NA	NA	NA	NA	NA	NA	NA	NA	NA
		30ng	NA	NA	NA	NA	NA	NA	NA	NA	NA	NA
			NA	NA	NA	NA	NA	NA	NA	NA	NA	NA
		45ng	NA	NA	NA	NA	NA	NA	NA	NA	NA	NA
			NA	NA	NA	NA	NA	NA	NA	NA	NA	NA
		60ng	NA	NA	NA	NA	NA	NA	NA	NA	NA	NA
			NA	NA	NA	NA	NA	NA	NA	NA	NA	NA

NA : Not amplified; NTC : No template control

<G12A>

PROMER	Sample		Ct									
G12A (Cy5)	NTC		NA	NA	NA	NA	NA	NA	NA	NA	NA	NA
			NA	NA	NA	NA	NA	NA	NA	NA	NA	NA
			NA	NA	NA	NA	NA	NA	NA	NA	NA	NA
			NA	NA	NA	NA	NA	NA	NA	NA	NA	NA
			NA	NA	NA	NA	NA	NA	NA	NA	NA	NA
			NA	NA	NA	NA	NA	NA	NA	NA	NA	NA
			NA	NA	NA	NA	NA	NA	NA	NA	NA	NA
			NA	NA	NA	NA	NA	NA	NA	NA	NA	NA
	WT (HEK293)	15ng	NA	NA	NA	NA	NA	NA	NA	NA	NA	NA
			NA	NA	NA	NA	NA	NA	NA	NA	NA	NA
		30ng	NA	NA	NA	NA	NA	NA	NA	NA	NA	NA
			NA	NA	NA	NA	NA	NA	NA	NA	NA	NA
		45ng	NA	NA	NA	NA	NA	NA	NA	NA	NA	NA
			NA	NA	NA	NA	NA	NA	NA	NA	NA	NA
		60ng	NA	NA	NA	NA	NA	NA	NA	NA	NA	NA
			NA	NA	NA	NA	NA	NA	NA	NA	NA	NA
	G12C (SW1573)	15ng	NA	NA	NA	NA	NA	NA	NA	NA	NA	NA
			NA	NA	NA	NA	NA	NA	NA	NA	NA	NA
		30ng	NA	NA	NA	NA	NA	NA	NA	NA	NA	NA
			NA	NA	NA	NA	NA	NA	NA	NA	NA	NA
		45ng	NA	NA	NA	NA	NA	NA	NA	NA	NA	NA
			NA	NA	NA	NA	NA	NA	NA	NA	NA	NA
		60ng	NA	NA	NA	NA	NA	NA	NA	NA	NA	NA
			NA	NA	NA	NA	NA	NA	NA	NA	NA	NA
	G12S (A549)	15ng	NA	NA	NA	NA	NA	NA	NA	NA	NA	NA
			NA	NA	NA	NA	NA	NA	NA	NA	NA	NA
		30ng	NA	NA	NA	NA	NA	NA	NA	NA	NA	NA
			NA	NA	NA	NA	NA	NA	NA	NA	NA	NA
		45ng	NA	NA	NA	NA	NA	NA	NA	NA	NA	NA
			NA	NA	NA	NA	NA	NA	NA	NA	NA	NA
		60ng	NA	NA	NA	NA	NA	NA	NA	NA	NA	NA
			NA	NA	NA	NA	NA	NA	NA	NA	NA	NA
	G12R (MDA-MB-134VI)	15ng	NA	NA	NA	NA	NA	NA	NA	NA	NA	NA
			NA	NA	NA	NA	NA	NA	NA	NA	NA	NA
		30ng	NA	NA	NA	NA	NA	NA	NA	NA	NA	NA
			NA	NA	NA	NA	NA	NA	NA	NA	NA	NA
		45ng	NA	NA	NA	NA	NA	NA	NA	NA	NA	NA
			NA	NA	NA	NA	NA	NA	NA	NA	NA	NA
		60ng	NA	NA	NA	NA	NA	NA	NA	NA	NA	NA
			NA	NA	NA	NA	NA	NA	NA	NA	NA	NA
	G12V (SW620)	15ng	NA	NA	NA	NA	NA	NA	NA	NA	NA	NA
			NA	NA	NA	NA	NA	NA	NA	NA	NA	NA
		30ng	NA	NA	NA	NA	NA	NA	NA	NA	NA	NA
			NA	NA	NA	NA	NA	NA	NA	NA	NA	NA
		45ng	NA	NA	NA	NA	NA	NA	NA	NA	NA	NA
			NA	NA	NA	NA	NA	NA	NA	NA	NA	NA

		60ng	NA	NA	NA	NA	NA	NA	NA	NA	NA	NA
			NA	NA	NA	NA	NA	NA	NA	NA	NA	NA
	G12A (NCI-H2009)	15ng	31.45	31.19	29.97	29.71	30.51	29.68	31.45	31.19	29.97	29.71
			32.46	31.67	30.42	30.29	31.05	30.16	32.46	31.67	30.42	30.29
		30ng	32.32	30.46	29.74	29.53	30.12	29.33	32.32	30.46	29.74	29.53
			32.13	30.31	29.47	29.26	29.67	29.20	32.13	30.31	29.47	29.26
		45ng	30.24	30.64	28.83	28.80	29.48	28.69	30.24	30.64	28.83	28.80
			30.88	30.63	28.87	28.96	30.31	28.95	30.88	30.63	28.87	28.96
		60ng	31.13	29.22	28.62	28.53	28.82	28.60	31.13	29.22	28.62	28.53
			30.59	28.60	27.99	28.05	28.04	28.17	30.59	28.60	27.99	28.05
	G12D SNU-C2B	15ng	NA	NA	NA	NA	NA	NA	NA	NA	NA	NA
			NA	NA	NA	NA	NA	NA	NA	NA	NA	NA
		30ng	NA	NA	NA	NA	NA	NA	NA	NA	NA	NA
			NA	NA	NA	NA	NA	NA	NA	NA	NA	NA
		45ng	NA	NA	NA	NA	NA	NA	NA	NA	NA	NA
			NA	NA	NA	NA	NA	NA	NA	NA	NA	NA
		60ng	NA	NA	NA	NA	NA	NA	NA	NA	NA	NA
			NA	NA	NA	NA	NA	NA	NA	NA	NA	NA

NA : Not amplified; NTC : No template control

<G12D>

PROMER	Sample		Ct									
G12D (FAM)	NTC		NA	NA	NA	NA	NA	NA	NA	NA	NA	NA
			NA	NA	NA	NA	NA	NA	NA	NA	39.83	NA
			NA	NA	NA	NA	NA	NA	NA	NA	NA	NA
			NA	NA	NA	NA	NA	NA	NA	NA	NA	NA
			NA	NA	NA	NA	NA	NA	NA	NA	NA	NA
			NA	NA	NA	NA	NA	NA	NA	NA	NA	NA
			NA	NA	NA	NA	NA	NA	NA	NA	NA	NA
			NA	NA	NA	NA	NA	NA	NA	NA	NA	39.71
	WT (HEK293)	15ng	NA	NA	NA	NA	NA	NA	NA	NA	NA	NA
			NA	NA	NA	NA	NA	NA	NA	NA	NA	NA
		30ng	NA	NA	NA	NA	NA	NA	NA	NA	NA	NA
			NA	NA	NA	NA	NA	NA	NA	NA	NA	NA
		45ng	39.81	NA	NA	NA	NA	NA	NA	NA	NA	NA
			NA	NA	NA	NA	NA	NA	NA	NA	NA	NA
		60ng	NA	NA	NA	NA	NA	NA	NA	NA	NA	NA
			38.06	NA	39.58	NA	NA	NA	NA	NA	NA	NA
	G12C (SW1573)	15ng	NA	NA	NA	NA	NA	NA	NA	NA	NA	NA
			NA	NA	NA	NA	NA	NA	NA	NA	NA	NA
		30ng	NA	NA	NA	NA	NA	NA	NA	NA	NA	NA
			NA	NA	NA	NA	NA	NA	NA	NA	NA	NA
		45ng	NA	NA	NA	NA	NA	NA	NA	NA	NA	NA
			NA	NA	NA	NA	NA	NA	NA	NA	NA	NA
		60ng	NA	NA	NA	NA	NA	NA	NA	NA	NA	NA
			NA	NA	NA	NA	NA	NA	NA	NA	NA	NA
	G12S (A549)	15ng	39.82	NA	NA	NA	NA	NA	NA	NA	NA	NA
			NA	NA	NA	NA	NA	NA	NA	NA	NA	NA
		30ng	NA	NA	NA	NA	NA	NA	NA	NA	NA	NA
			NA	NA	NA	NA	NA	NA	NA	NA	NA	NA
		45ng	NA	NA	NA	NA	NA	NA	NA	NA	NA	NA
			NA	NA	NA	NA	NA	NA	NA	NA	NA	NA
		60ng	NA	NA	NA	NA	NA	NA	NA	NA	NA	NA
			NA	NA	NA	NA	NA	NA	NA	NA	NA	NA
	G12R (MDA-MB-134VI)	15ng	NA	NA	NA	NA	NA	NA	NA	NA	NA	NA
			NA	NA	NA	NA	NA	NA	NA	NA	NA	NA
		30ng	NA	NA	NA	NA	NA	NA	NA	NA	NA	NA
			NA	NA	NA	NA	NA	NA	NA	NA	NA	NA
		45ng	NA	NA	NA	NA	NA	NA	NA	NA	NA	NA
			NA	NA	NA	NA	NA	NA	NA	NA	NA	NA
		60ng	NA	NA	NA	NA	NA	NA	NA	NA	NA	NA
			NA	NA	NA	NA	NA	NA	NA	NA	NA	NA
	G12V (SW620)	15ng	NA	NA	NA	NA	NA	NA	NA	NA	NA	NA
			NA	NA	NA	NA	NA	NA	NA	NA	NA	NA
		30ng	NA	NA	NA	NA	NA	NA	NA	NA	NA	NA
			NA	NA	NA	NA	NA	NA	NA	NA	NA	NA
		45ng	NA	NA	NA	NA	NA	NA	NA	NA	NA	NA
			38.81	NA	NA	NA	NA	NA	NA	NA	NA	NA

		60ng	NA	NA	NA	NA	NA	NA	NA	NA	NA	NA
			NA	NA	NA	NA	NA	NA	NA	NA	NA	NA
	G12A (NCI-H2009)	15ng	NA	NA	NA	39.86	NA	NA	NA	NA	NA	NA
			NA	NA	NA	NA	NA	NA	NA	NA	NA	NA
		30ng	NA	NA	NA	39.30	NA	NA	NA	NA	NA	NA
			NA	NA	NA	NA	NA	NA	NA	NA	NA	NA
		45ng	NA	NA	NA	NA	NA	NA	NA	NA	NA	NA
			NA	NA	NA	NA	NA	NA	NA	NA	NA	NA
		60ng	NA	NA	NA	NA	NA	NA	NA	NA	NA	NA
			NA	NA	NA	39.81	39.61	NA	NA	NA	NA	NA
	G12D SNU-C2B	15ng	30.04	29.98	29.75	29.70	29.77	29.73	30.04	29.98	29.75	29.70
			30.09	29.86	29.77	30.10	29.76	29.83	30.09	29.86	29.77	30.10
		30ng	29.47	29.35	29.17	29.35	29.15	29.22	29.47	29.35	29.17	29.35
			29.44	29.23	29.24	29.15	28.95	29.19	29.44	29.23	29.24	29.15
		45ng	29.35	29.27	28.71	29.02	28.92	29.05	29.35	29.27	28.71	29.02
			29.29	29.04	28.89	28.89	29.09	28.90	29.29	29.04	28.89	28.89
		60ng	29.10	29.02	29.02	28.76	28.83	28.77	29.10	29.02	29.02	28.76
			29.13	28.81	28.48	28.53	28.68	28.58	29.13	28.81	28.48	28.53

NA : Not amplified; NTC : No template control

Supplementary Table S9. Composition of template used in the multiplexed PROMER PCR.

Testing Genotype	Genomic DNA (Copy Number)		
G12S/C/R	Testing Copy #	MIA PaCa-2 (G12S), A549 (G12C), or MDA-MB-134VI(G/12R)	HEK-293 (WT)
	$1x10^4$	$1x10^4$	0
	$1x10^3$	$1x10^3$	$1x10^5$
	$1x10^2$	$1x10^2$	$1x10^5$
	$1x10^1$	$1x10^1$	$1x10^5$
	$0.5x10^1$	$0.5x10^1$	$1x10^5$
	$1x10^0$	$1x10^0$	$1x10^5$
	0	0	$1x10^5$
G12V/D/A	Testing Copy #	SW620 (G12V), Panc-1 (G12D), or NCI-H2009 (G/12A)	HEK-293 (WT)
	$1x10^4$	$1x10^4$	0
	$1x10^3$	$1x10^3$	$1x10^5$
	$1x10^2$	$1x10^2$	$1x10^5$
	$1x10^1$	$1x10^1$	$1x10^5$
	$0.5x10^1$	$0.5x10^1$	$1x10^5$
	$1x10^0$	$1x10^0$	$1x10^5$
	0	0	$1x10^5$

Supplementary Table S10. Extracted cfDNA from the animals' plasma injection of the indicated cells. Each copy number is calculated based on Supplementary Table S7.

NCI-H358 (G12C)

Mouse Number	Tumor size			cfDNA	PROMER PCR		TaqMan PCR	
	W (mm)	L (mm)	Volume (mm^3)	Conc. (pg/μL)	Ct mean	Copy# per cfDNA (1ng)	Ct mean	Copy# per cfDNA (1ng)
H358-1	6.8	10.0	228.9	105.13	34.96	16.6	33.21	18.0
H358-2	7.9	8.5	265.2	98.89	30.68	321.6	29.28	219.8
H358-3	6.8	7.5	173.2	160.27	34.28	17.5	33.03	13.3
H358-4	10.6	11.9	668.8	292.93	30.97	89.1	29.70	57.1
H358-5	12.2	11.3	837.4	1338.23	24.98	1117.4	22.20	1317.3

A549 (G12S)

Mouse Number	Tumor size			cfDNA	PROMER PCR		TaqMan PCR	
	W (mm)	L (mm)	Volume (mm^3)	Conc. (pg/μL)	Ct mean	Copy# per cfDNA (1ng)	Ct mean	Copy# per cfDNA (1ng)
A549-1	5.8	8.4	141.1	174.66	36.05	3.1	ND	ND
A549-2	4.7	8.4	90.7	28.28	37.09	10.6	34.21	32.0
A549-3	3.5	4.5	26.7	17.09	ND	ND	ND	ND
A549-4	5.7	5.9	94.8	65.12	35.59	12.3	34.31	13.0
A549-5	6.4	7.4	151.6	197.53	37.30	1.3	34.36	4.2

SW620 (G12V)

Mouse Number	Tumor size			cfDNA	PROMER PCR		TaqMan PCR	
	W (mm)	L (mm)	Volume (mm^3)	Conc. (pg/μL)	Ct mean	Copy# per cfDNA (1ng)	Ct mean	Copy# per cfDNA (1ng)
SW620-1	10.2	12.2	634.6	357.16	30.76	99.5	29.63	32.1
SW620-2	8.4	9.7	342.2	717.63	29.73	95.8	29.37	18.3
SW620-3	2.5	2.6	8.1	156.75	35.05	14.4	32.06	21.0
SW620-4	9.1	10.5	430.8	260.42	32.30	50.5	30.68	25.8
SW620-5	9.5	11.5	512.6	206.00	32.59	53.4	30.94	28.4
SW620-5	9.0	11.4	458.4	189.38	32.10	79.2	30.36	41.6
SW620-6	10.2	11.2	586.5	217.03	32.71	37.1	32.45	12.4

SNU-C2B (G12D)

Mouse Number	Tumor size			cfDNA	PROMER PCR		TaqMan PCR	
	W (mm)	L (mm)	Volume (mm^3)	Conc. (pg/μL)	Ct mean	Copy# per cfDNA (1ng)	Ct mean	Copy# per cfDNA (1ng)
C2B-1	6.3	8.3	164.7	82.12	34.51	81.6	33.14	29.2

C2B-2	8.8	8.5	323.5	78.80	35.76	35.5	35.35	7.0
C2B-3	7.9	10.2	318.3	92.79	34.83	58.2	33.66	18.3
C2B-4	5.0	9.4	117.5	514.1	35.38	7.1	40.39	0.0
C2B-5	7.1	7.3	184.0	252.31	34.59	25.4	31.90	21.6
C2B-6	5.2	6.3	85.9	157.37	37.72	4.4	34.02	8.6
C2B-7	8.7	14.5	549.4	438.96	32.57	58.9	31.33	18.1
C2B-8	12.5	13.1	1023.4	1018.07	29.31	244.3	27.59	93.1
C2B-9	10.6	13.6	760.0	470.81	31.46	118.6	30.30	33.5

Panc-1 (G12D)

Mouse Number	Tumor size			cfDNA	PROMER PCR		TaqMan PCR	
	W (mm)	L (mm)	Volume (mm^3)	Conc. (pg/μL)	Ct mean	Copy# per cfDNA (1ng)	Ct mean	Copy# per cfDNA (1ng)
Panc-1-1	8.6	9.3	343.9	88.51	35.73	32.8	33.39	23.2
Panc-1-2	8.5	8.7	314.3	136.70	34.87	38.0	31.73	44.6
Panc-1-3	10.0	10.6	529.0	332.62	32.16	103.4	29.04	109.3

NCI-H2009 (G12A)

Mouse Number	Tumor size			cfDNA	PROMER PCR		TaqMan PCR	
	W (mm)	L (mm)	Volume (mm^3)	Conc. (pg/μL)	Ct mean	Copy# per cfDNA (1ng)	Ct mean	Copy# per cfDNA (1ng)
H2009-1	11.0	11.4	685.2	354.10	37.27	4.0	32.19	11.8
H2009-2	8.5	10.0	361.3	370.39	40.68	0.3	32.85	7.3
H2009-3	8.7	10.3	389.8	62.68	34.93	119.7	31.84	83.8

SW1116 (G12A)

Mouse Number	Tumor size			cfDNA	PROMER PCR		TaqMan PCR	
	W (mm)	L (mm)	Volume (mm^3)	Conc. (pg/μL)	Ct mean	Copy# per cfDNA (1ng)	Ct mean	Copy# per cfDNA (1ng)
SW1116-1	8.4	9.7	342.2	98.95	36.04	33.9	ND	ND
SW1116-2	12.3	12.7	951.4	224.38	34.44	47.7	37.20	0.7

HCT-15 (G13D)

Mouse Number	Tumor size			cfDNA	PROMER PCR		TaqMan PCR	
	W (mm)	L (mm)	Volume (mm^3)	Conc. (pg/μL)	Ct mean	Copy# per cfDNA (1ng)	Ct mean	Copy# per cfDNA (1ng)
HCT-15-1	8.8	11.5	448.6	54.45	35.67	162.3	32.56	198.1
HCT-15-2	6.6	7.3	159.0	170.65	37.24	19.6	35.40	14.9

HCT-15-3	5.7	8.4	136.5	108.13	39.98	5.7	ND	ND

MDA-MB-231 (G13D)

Mouse Number	Tumor size			cfDNA	PROMER PCR		TaqMan PCR	
	W (mm)	L (mm)	Volume (mm^3)	Conc. (pg/μL)	Ct mean	Copy# per cfDNA (1ng)	Ct mean	Copy# per cfDNA (1ng)
231-1	11.8	11.9	823.6	1019.22	33.56	31.9	30.84	25.3
231-2	10.6	12.8	719.1	278.41	35.19	42.6	31.59	63.2
231-3	8.9	11.8	467.3	1197.53	32.42	55.0	29.06	53.1
231-4	10.8	13.4	781.5	614.39	30.69	311.8	27.38	244.1
231-5	13.1	16.1	1381.5	1701.79	31.15	84.8	27.91	67.1

NCI-H460 (Q61H)

Mouse Number	Tumor size			cfDNA	PROMER PCR		TaqMan PCR	
	W (mm)	L (mm)	Volume (mm^3)	Conc. (pg/μL)	Ct mean	Copy# per cfDNA (1ng)	Ct mean	Copy# per cfDNA (1ng)
H460-1	10.8	13.6	784.7	705.85	29.10	521.5	25.53	178.6
H460-2	8.9	12.6	499.0	541.14	30.72	237.9	27.10	100.3
H460-3	4.1	5.9	49.6	296.18	33.56	68.4	29.89	115.8
H460-4	8.2	12.2	410.2	670.04	29.97	311.6	26.76	41.4
H460-5	9.0	9.4	380.7	460.31	30.29	369.4	26.87	39.7

Supplementary Table S11. PROMER PCR Results with lower annealing and extension temperature. Genomic DNA of indicated cell lines were used as PCR templates. NTC: No template control, NA: Not amplified.

MIA PaCa-2 (G12C)

Annealing & Extension Temperature (°C)	Template (33.4 ng)	Ct
56	SW1573 (G12C)	26.49
		26.49
		26.42
	HEK-293 (WT)	NA
		NA
		NA
	NTC	NA
		NA
		NA
58	SW1573 (G12C)	26.53
		26.67
		26.61
	HEK-293 (WT)	NA
		NA
		NA
	NTC	NA
		NA
		NA
60	SW1573 (G12C)	26.98
		26.88
		26.78
	HEK-293 (WT)	NA
		NA
		NA
	NTC	NA
		NA
		NA

A549 (G12S)

Annealing & Extension Temperature (°C)	Template (33.4 ng)	Ct
56	A549 (G12S)	24.50
		24.64
		24.67
	HEK-293 (WT)	35.72
		35.81
		35.65
	NTC	NA
		NA
		NA
58	A549 (G12S)	24.74
		24.54

		24.86
	HEK-293 (WT)	35.43
		36.25
		35.65
	NTC	NA
		NA
		NA
60	A549 (G12S)	25.03
		25.09
		24.86
	HEK-293 (WT)	37.40
		36.54
		37.41
	NTC	NA
		NA
		NA

MDA-MB-134VI (G12R)

Annealing & Extension Temperature (°C)	Template (33.4 ng)	Ct
56	MDA-MB-134VI (G12R)	25.92
		26.08
		26.03
	HEK-293 (WT)	NA
		NA
		NA
	NTC	NA
		NA
		NA
58	MDA-MB-134VI (G12R)	26.53
		26.67
		26.61
	HEK-293 (WT)	NA
		NA
		NA
	NTC	NA
		NA
		NA
60	MDA-MB-134VI (G12R)	25.29
		25.32
		25.45
	HEK-293 (WT)	NA
		39.91
		NA
	NTC	NA
		NA
		NA

SW620 (G12V)

Annealing & Extension Temperature (°C)	Template (33.4 ng)	Ct
56	SW620 (G12V)	25.51
		25.49
		25.52
	HEK-293 (WT)	NA
		NA
		NA
	NTC	NA
		NA
		NA
58	SW620 (G12V)	25.24
		25.21
		25.26
	HEK-293 (WT)	NA
		NA
		NA
	NTC	NA
		NA
		NA
60	SW620 (G12V)	24.98
		24.95
		24.92
	HEK-293 (WT)	NA
		NA
		NA
	NTC	NA
		NA
		NA

SNU-C2B (G12D)

Annealing & Extension Temperature (°C)	Template (33.4 ng)	Ct
56	SNU-C2B (G12V)	26.39
		26.49
		26.58
	HEK-293 (WT)	37.43
		37.07
		36.80
	NTC	NA
		NA
		NA
58	SNU-C2B (G12V)	26.63
		26.51
		26.72
	HEK-293 (WT)	37.53
		37.86
		37.92
	NTC	NA
		NA

		NA
60	SNU-C2B (G12V)	26.82
		26.86
		26.74
	HEK-293 (WT)	38.32
		37.27
		37.07
	NTC	NA
		NA
		NA

NCI-H2009 (G12A)

Annealing & Extension Temperature (°C)	Template (33.4 ng)	Ct
56	NCI-H2009 (G12A)	24.62
		24.57
		24.65
	HEK-293 (WT)	NA
		NA
		NA
	NTC	NA
		NA
		NA
58	NCI-H2009 (G12A)	24.48
		24.48
		24.43
	HEK-293 (WT)	NA
		NA
		NA
	NTC	NA
		NA
		NA
60	NCI-H2009 (G12A)	24.41
		24.43
		24.39
	HEK-293 (WT)	NA
		NA
		NA
	NTC	NA
		NA
		NA

Supplementary Figure S1. cfDNA measured using Agilent 2100 BioAnalyzer.

G12C

NCI-H358-1

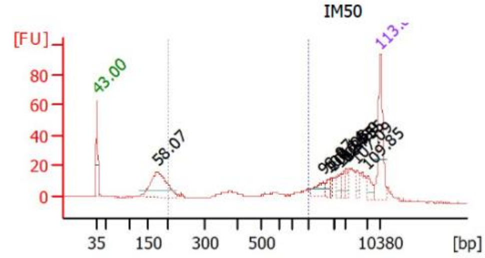

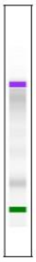

Peak table for sample 9 : **IM50**

Peak		Size [bp]	Conc. [pg/µl]	Molarity [pmol/l]	Observations
1	◀	35	125.00	5,411.3	Lower Marker
2		175	175.21	1,519.4	
3		1,589	18.79	17.9	
4		1,797	8.32	7.0	
5		1,950	6.22	4.8	
6		2,511	9.80	5.9	
7		2,815	11.40	6.1	
8		3,074	9.63	4.7	
9		5,071	12.10	3.6	
10		7,300	15.07	3.1	
11	▶	10,380	75.00	10.9	Upper Marker

Overall Results for sample 9 : **IM50**

Number of peaks found:	9
Noise:	0.2
Corr. Area 1:	127.1

NCI-H358-2

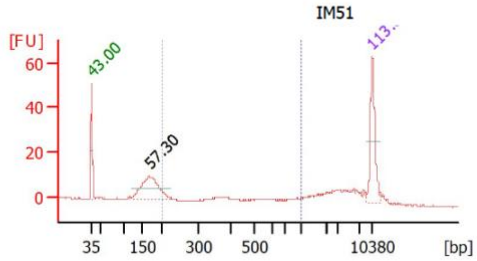

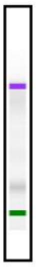

Peak table for sample 10 : **IM51**

Peak		Size [bp]	Conc. [pg/µl]	Molarity [pmol/l]	Observations
1	◀	35	125.00	5,411.3	Lower Marker
2		167	160.69	1,461.6	
3	▶	10,380	75.00	10.9	Upper Marker

Overall Results for sample 10 : **IM51**

Number of peaks found:	1
Noise:	0.1
Corr. Area 1:	41.5

NCI-H358-3

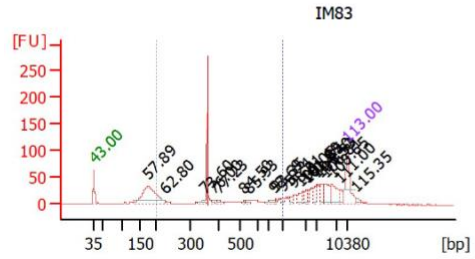

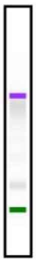

Peak table for sample 6 : **IM83**

Peak		Size [bp]	Conc. [pg/µl]	Molarity [pmol/l]	Observations
1	◀	35	125.00	5,411.3	Lower Marker
2		173	267.11	2,333.5	
3		226	5.38	36.0	
4		350	15.84	68.5	
5		380	7.18	28.6	
6		396	11.11	42.5	
7		525	4.09	11.8	
8		556	7.14	19.5	
9		815	14.78	27.5	
10		864	4.40	7.7	
11		1,008	7.30	11.0	
12		1,454	9.43	9.8	
13		1,784	16.48	14.0	
14		2,093	10.57	7.7	
15		2,141	10.34	7.3	
16		2,948	18.66	9.6	
17		3,750	23.35	9.4	
18		4,751	42.26	13.5	
19		6,191	42.66	10.4	
20		8,345	13.63	2.5	
21	▶	10,380	75.00	10.9	Upper Marker

Overall Results for sample 6 : **IM83**

Number of peaks found:	20
Noise:	0.4
Corr. Area 1:	321.4

NCI-H358-4

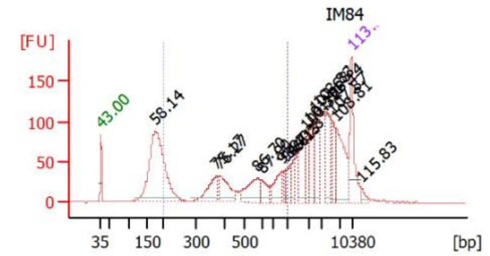

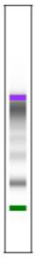

Peak table for sample 7 : IM84

Peak	Size [bp]	Conc. [pg/µl]	Molarity [pmol/l]	Observations
1	35	125.00	5,411.3	Lower Marker
2	176	476.01	4,095.1	
3	370	64.07	262.4	
4	384	65.35	258.0	
5	572	65.66	173.8	
6	598	24.38	61.8	
7	868	43.70	76.2	
8	957	14.76	23.4	
9	1,151	27.03	35.6	
10	1,337	20.10	22.8	
11	1,827	59.51	49.4	
12	1,953	28.04	21.8	
13	2,404	48.03	30.3	
14	2,767	43.28	23.7	
15	4,225	45.50	16.3	
16	5,577	28.33	7.7	
17	6,315	84.38	20.2	
18	10,380	75.00	10.9	Upper Marker

Overall Results for sample 7 : IM84

Number of peaks found: 17
Noise: 0.3
Corr. Area 1: 916.7

NCI-H358-5

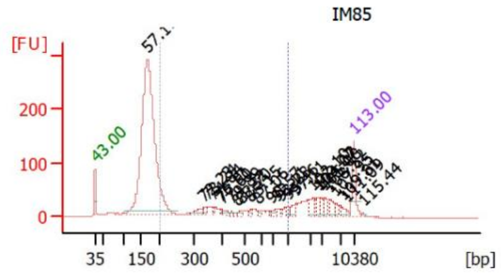

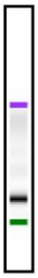

Peak table for sample 8 : IM85

Peak	Size [bp]	Conc. [pg/µl]	Molarity [pmol/l]	Observations
1	35	125.00	5,411.3	Lower Marker
2	166	2,125.42	19,401.9	
3	333	36.98	168.1	
4	346	15.29	67.0	
5	366	12.48	51.7	
6	392	9.24	35.8	
7	400	15.75	59.6	
8	440	5.12	17.6	
9	465	4.26	13.9	
10	490	10.05	31.1	
11	539	9.80	27.5	
12	580	10.21	26.7	
13	682	5.80	12.9	
14	773	12.92	25.3	
15	859	7.08	12.5	
16	1,043	11.27	16.4	
17	1,309	17.63	20.4	
18	2,223	21.51	14.7	
19	2,591	19.45	11.4	
20	2,843	17.95	9.6	
21	3,399	18.53	8.3	
22	4,522	22.53	7.5	
23	5,644	14.25	3.8	
24	7,129	11.36	2.4	
25	8,429	6.02	1.1	

Overall Results for sample 8 : IM85

Number of peaks found: 25
Noise: 0.4
Corr. Area 1: 661.2

NCI-H358-6

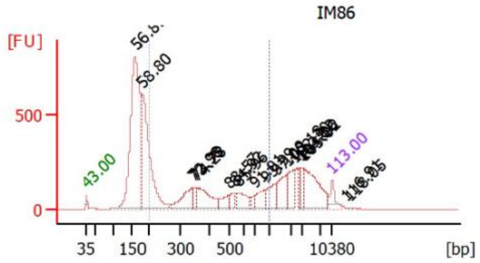

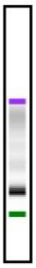

Peak table for sample 9 : IM86

Peak	Size [bp]	Conc. [pg/µl]	Molarity [pmol/l]	Observations
1	35	125.00	5,411.3	Lower Marker
2	162	2,738.30	25,560.6	
3	183	1,550.68	12,827.3	
4	343	270.66	1,197.1	
5	353	58.75	252.3	
6	359	336.77	1,421.9	
7	506	110.16	330.1	
8	535	55.16	156.4	
9	557	131.67	358.5	
10	696	45.32	98.6	
11	912	115.32	191.7	
12	1,306	146.79	170.3	
13	1,805	175.08	147.0	
14	2,285	146.14	96.9	
15	2,764	65.94	36.1	
16	2,901	50.43	26.3	
17	3,350	346.11	156.5	
18	10,380	75.00	10.9	Upper Marker

Overall Results for sample 9 : IM86

Number of peaks found: 18
Noise: 0.4
Corr. Area 1: 3,817.2

G12S
A549-1

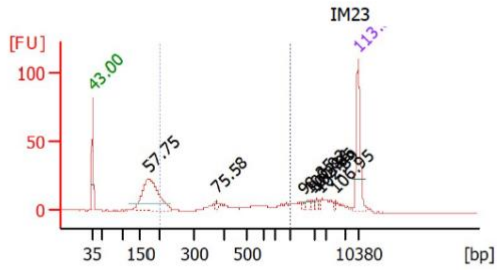

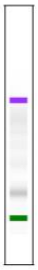

Peak table for sample 8 : IM23

Peak		Size [bp]	Conc. [pg/µl]	Molarity [pmol/l]	Observations
1	◀	35	125.00	5,411.3	Lower Marker
2		171	232.88	2,060.8	
3		373	3.83	15.5	
4		1,507	2.35	2.4	
5		1,779	6.35	5.4	
6		1,916	3.34	2.6	
7		2,221	4.28	2.9	
8		2,492	3.16	1.9	
9		4,946	2.76	0.8	
10	▶	10,380	75.00	10.9	Upper Marker

Overall Results for sample 8 : IM23

Number of peaks found:	8
Noise:	0.2
Corr. Area 1:	152.7

A549-2

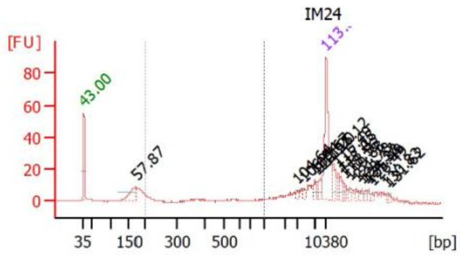

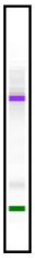

Peak table for sample 9 : IM24

Peak		Size [bp]	Conc. [pg/µl]	Molarity [pmol/l]	Observations
1	◀	35	125.00	5,411.3	Lower Marker
2		172	37.71	331.3	
3		3,145	2.30	1.1	
4		4,628	3.58	1.2	
5		7,075	4.41	0.9	
6		7,914	5.29	1.0	
7		8,596	5.49	1.0	
8	▶	10,380	75.00	10.9	Upper Marker
9		12,478	0.00	0.0	
10		13,842	0.00	0.0	
11		14,524	0.00	0.0	
12		15,364	0.00	0.0	
13		15,888	0.00	0.0	
14		17,200	0.00	0.0	
15		19,088	0.00	0.0	
16		20,662	0.00	0.0	
17		21,607	0.00	0.0	
18		22,761	0.00	0.0	
19		23,758	0.00	0.0	
20		28,059	0.00	0.0	
21		28,846	0.00	0.0	

Overall Results for sample 9 : IM24

Number of peaks found:	19
Noise:	0.2
Corr. Area 1:	53.5

A549-3

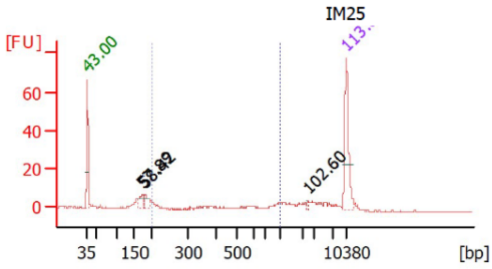

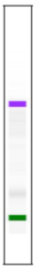

Peak table for sample 10 : IM25

Peak		Size [bp]	Conc. [pg/µl]	Molarity [pmol/l]	Observations
1	◀	35	125.00	5,411.3	Lower Marker
2		173	22.79	200.0	
3		178	19.92	169.2	
4		2,397	2.69	1.7	
5	▶	10,380	75.00	10.9	Upper Marker

Overall Results for sample 10 : IM25

Number of peaks found:	3
Noise:	0.2
Corr. Area 1:	28.4

A549-4

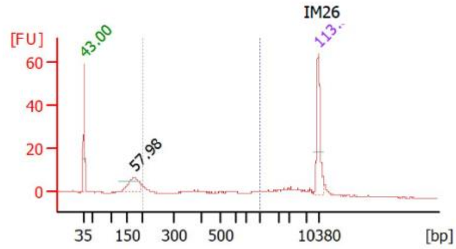

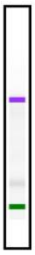

Peak table for sample 6 : IM26

Peak		Size [bp]	Conc. [pg/µl]	Molarity [pmol/l]	Observations
1	◀	35	125.00	5,411.3	Lower Marker
2		172	86.82	767.0	
3	▶	10,380	75.00	10.9	Upper Marker

Overall Results for sample 6 : IM26

Number of peaks found:	1
Noise:	0.1
Corr. Area 1:	40.4

A549-5

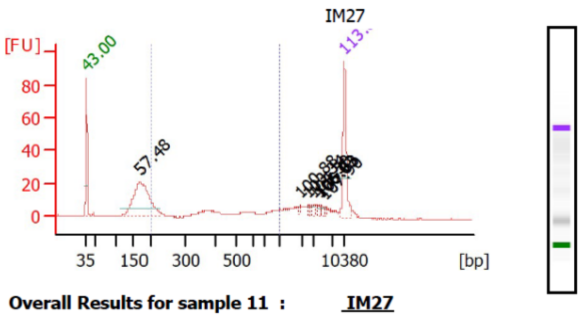

Overall Results for sample 11 : **IM27**

Number of peaks found:	7
Noise:	0.3
Corr. Area 1:	119.8

Peak table for sample 11 : **IM27**

Peak		Size [bp]	Conc. [pg/µl]	Molarity [pmol/l]	Observations
1	◀	35	125.00	5,411.3	Lower Marker
2		168	263.37	2,369.8	
3		1,919	2.87	2.3	
4		2,640	3.83	2.2	
5		3,097	4.07	2.0	
6		4,225	3.23	1.2	
7		4,851	4.67	1.5	
8		5,686	3.37	0.9	
9	▶	10,380	75.00	10.9	Upper Marker

G12V

SW620-1

Peak table for sample 3 : **IM113**

Peak		Size [bp]	Conc. [pg/µl]	Molarity [pmol/l]	Observations
1	◀	35	125.00	5,411.3	Lower Marker
2		170	595.27	5,300.6	
3		377	52.98	213.0	
4		384	22.77	89.9	
5		1,646	5.78	5.3	
6		1,825	7.80	6.5	
7		1,989	10.96	8.3	
8		2,683	8.94	5.1	
9		3,803	15.84	6.3	
10		4,784	18.30	5.8	
11		6,480	12.79	3.0	
12		8,255	4.85	0.9	
13		9,032	4.43	0.7	
14	▶	10,380	75.00	10.9	Upper Marker

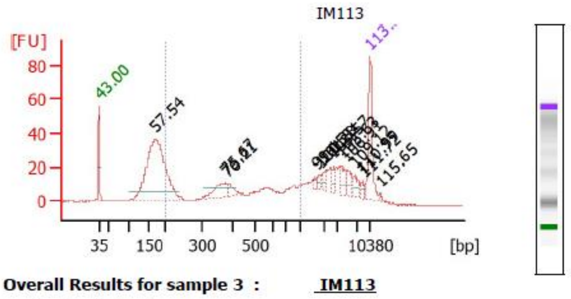

Overall Results for sample 3 : **IM113**

Number of peaks found:	13
Noise:	0.2
Corr. Area 1:	328.9

SW620-2

Peak table for sample 4 : **IM114**

Peak		Size [bp]	Conc. [pg/µl]	Molarity [pmol/l]	Observations
1	◀	35	125.00	5,411.3	Lower Marker
2		173	1,435.25	12,577.2	
3		374	155.29	629.6	
4		384	94.25	372.1	
5		515	52.64	154.8	
6		537	17.36	49.0	
7		565	15.26	40.9	
8		582	46.13	120.0	
9		836	38.26	69.4	
10		888	10.47	17.9	
11		1,090	12.20	17.0	
12		1,273	17.03	20.3	
13		1,656	44.55	40.8	
14		1,824	22.14	18.4	
15		1,967	22.52	17.3	
16		2,752	47.50	26.2	
17		2,956	22.35	11.5	
18		3,627	24.24	10.1	
19		4,515	30.48	10.2	
20		5,365	30.13	8.5	
21		6,330	49.86	11.9	
22	▶	10,380	75.00	10.9	Upper Marker

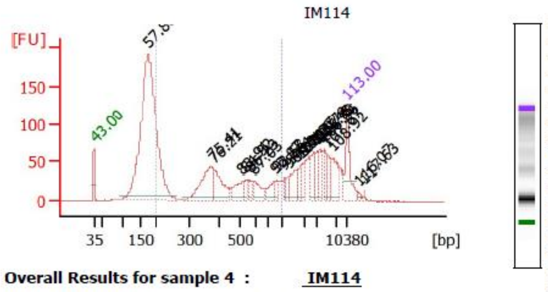

Overall Results for sample 4 : **IM114**

Number of peaks found:	22
Noise:	0.2
Corr. Area 1:	1,077.5

SW620-3

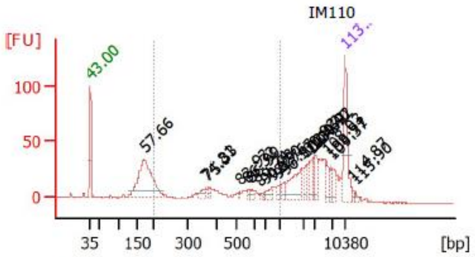

Overall Results for sample 11 : IM110

Number of peaks found:	21
Noise:	0.3
Corr. Area 1:	264.5

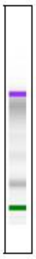

Peak table for sample 11 : IM110

Peak		Size [bp]	Conc. [pg/µl]	Molarity [pmol/l]	Observations
1	◀	35	125.00	5,411.3	Lower Marker
2		170	254.72	2,267.3	
3		364	14.52	60.4	
4		371	7.88	32.2	
5		513	3.40	10.1	
6		563	5.66	15.2	
7		578	7.86	20.6	
8		654	2.85	6.6	
9		694	3.56	7.8	
10		829	11.84	21.6	
11		967	7.95	12.5	
12		1,204	10.39	13.1	
13		1,906	50.28	40.0	
14		2,213	10.91	7.5	
15		2,711	16.57	9.3	
16		2,960	12.75	6.5	
17		3,310	16.10	7.4	
18		5,760	15.08	4.0	
19		6,454	9.95	2.3	
20		6,821	19.10	4.2	
21	▶	10,380	75.00	10.9	Upper Marker

SW620-4

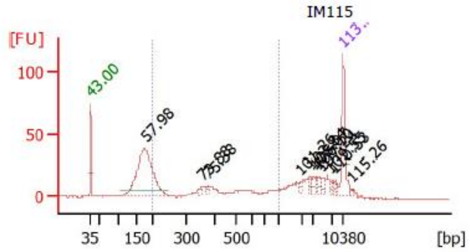

Overall Results for sample 5 : IM115

Number of peaks found:	11
Noise:	0.3
Corr. Area 1:	240.0

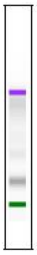

Peak table for sample 5 : IM115

Peak		Size [bp]	Conc. [pg/µl]	Molarity [pmol/l]	Observations
1	◀	35	125.00	5,411.3	Lower Marker
2		175	423.19	3,666.7	
3		355	7.19	30.7	
4		373	7.37	29.9	
5		1,982	4.41	3.4	
6		2,767	6.38	3.5	
7		3,375	10.49	4.7	
8		4,165	6.73	2.4	
9		5,034	10.86	3.3	
10		6,812	6.73	1.5	
11		7,773	6.15	1.2	
12	▶	10,380	75.00	10.9	Upper Marker

SW620-5

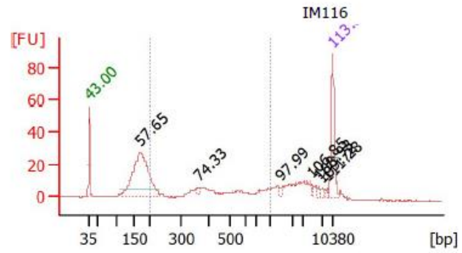

Overall Results for sample 6 : IM116

Number of peaks found:	7
Noise:	0.2
Corr. Area 1:	181.4

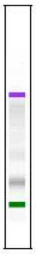

Peak table for sample 6 : IM116

Peak		Size [bp]	Conc. [pg/µl]	Molarity [pmol/l]	Observations
1	◀	35	125.00	5,411.3	Lower Marker
2		171	412.00	3,644.2	
3		360	7.44	31.3	
4		1,463	4.02	4.2	
5		4,738	5.39	1.7	
6		6,338	4.50	1.1	
7		6,938	3.90	0.9	
8		8,565	2.48	0.4	
9	▶	10,380	75.00	10.9	Upper Marker

SW620-6

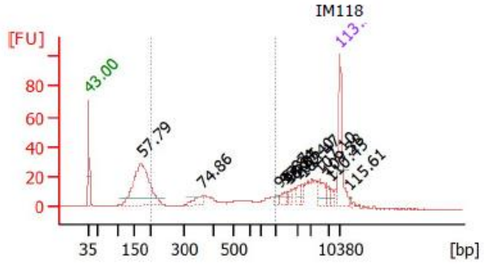

Overall Results for sample 8 : IM118

Number of peaks found:	12
Noise:	0.2
Corr. Area 1:	195.4

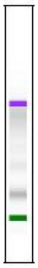

Peak table for sample 8 : IM118

Peak		Size [bp]	Conc. [pg/µl]	Molarity [pmol/l]	Observations
1	◀	35	125.00	5,411.3	Lower Marker
2		173	315.64	2,767.3	
3		367	26.44	109.2	
4		1,127	6.33	8.5	
5		1,439	8.94	9.4	
6		1,523	4.62	4.6	
7		1,633	5.63	5.2	
8		2,007	9.46	7.1	
9		2,339	6.82	4.4	
10		4,927	21.81	6.7	
11		6,683	8.86	2.0	
12		7,686	7.01	1.4	
13	▶	10,380	75.00	10.9	Upper Marker

SW620-7

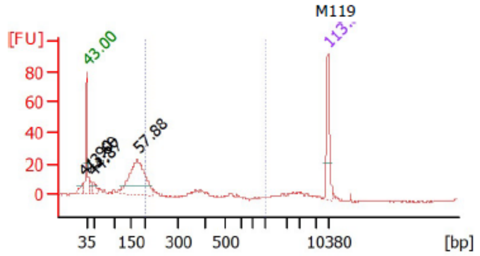

Overall Results for sample 9 : M119

Number of peaks found: 4
Noise: 0.3
Corr. Area 1: 55.6

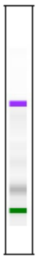

Peak table for sample 9 : M119

Peak		Size [bp]	Conc. [pg/µl]	Molarity [pmol/l]	Observations
1		31	0.00	0.0	
2	◀	35	125.00	5,411.3	Lower Marker
3		39	40.78	1,589.8	
4		47	39.89	1,287.3	
5		174	352.67	3,074.5	
6	▶	10,380	75.00	10.9	Upper Marker

G12D

SNU-C2B -1

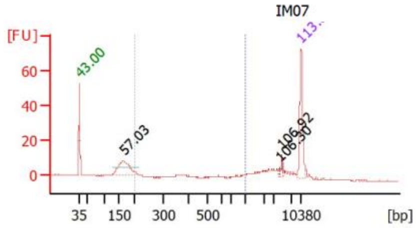

Overall Results for sample 6 : IM07

Number of peaks found: 3
Noise: 0.1
Corr. Area 1: 22.1

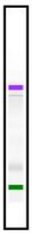

Peak table for sample 6 : IM07

Peak		Size [bp]	Conc. [pg/µl]	Molarity [pmol/l]	Observations
1	◀	35	125.00	5,411.3	Lower Marker
2		165	136.86	1,257.9	
3		4,332	3.76	1.3	
4		4,824	7.14	2.2	
5	▶	10,380	75.00	10.9	Upper Marker

SNU-C2B-2

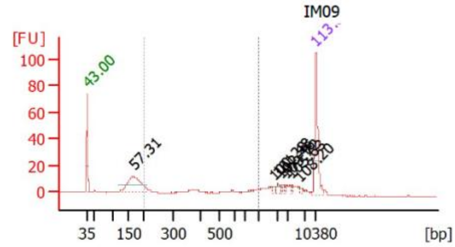

Overall Results for sample 8 : IM09

Number of peaks found: 7
Noise: 0.2
Corr. Area 1: 65.3

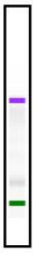

Peak table for sample 8 : IM09

Peak		Size [bp]	Conc. [pg/µl]	Molarity [pmol/l]	Observations
1	◀	35	125.00	5,411.3	Lower Marker
2		168	131.33	1,186.2	
3		1,808	2.72	2.3	
4		2,002	6.45	4.9	
5		2,316	3.11	2.0	
6		2,716	3.18	1.8	
7		3,805	3.59	1.4	
8		5,829	2.51	0.7	
9	▶	10,380	75.00	10.9	Upper Marker

SNU-C2B-3

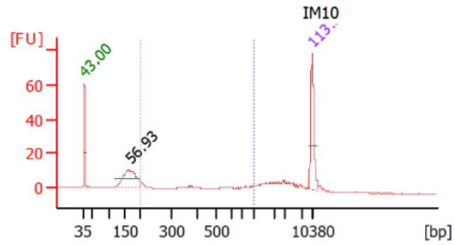

Overall Results for sample 9 : IM10

Number of peaks found: 1
Noise: 0.2
Corr. Area 1: 51.5

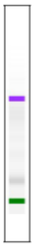

Peak table for sample 9 : IM10

Peak		Size [bp]	Conc. [pg/µl]	Molarity [pmol/l]	Observations
1	◀	35	125.00	5,411.3	Lower Marker
2		164	150.78	1,394.7	
3	▶	10,380	75.00	10.9	Upper Marker

SNU-C2B-4

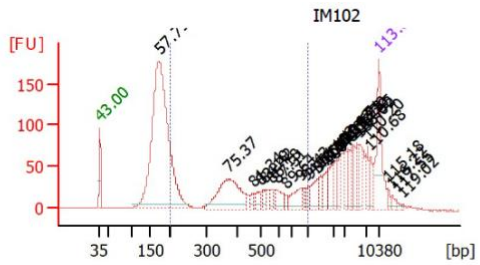

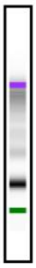

Peak table for sample 3 : **IM102**

Peak		Size [bp]	Conc. [pg/µl]	Molarity [pmol/l]	Observations
1	◀	35	125.00	5,411.3	Lower Marker
2		172	856.84	7,568.5	
3		371	140.60	573.7	
4		465	7.75	25.3	
5		497	11.37	34.6	
6		523	9.52	27.6	
7		560	8.51	23.0	
8		585	21.26	55.1	
9		651	5.59	13.0	
10		868	28.80	50.3	
11		956	6.26	9.9	
12		1,041	7.63	11.1	
13		1,416	25.71	27.5	
14		1,508	10.87	10.9	
15		1,776	20.02	17.1	
16		1,952	19.77	15.3	
17		2,329	22.40	14.6	
18		2,572	14.76	8.7	
19		3,144	17.53	8.5	
20		3,555	20.76	8.8	
21		4,414	13.78	4.7	
22		4,787	14.97	4.7	
23		5,384	14.46	4.1	
24		5,758	23.81	6.3	
25		7,600	11.91	2.4	

Overall Results for sample 3 : **IM102**

Number of peaks found:	29
Noise:	0.5
Corr. Area 1:	965.5

SNU-C2B-5

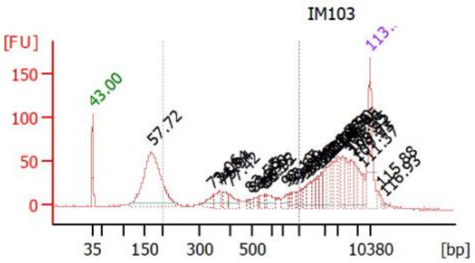

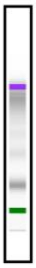

Peak table for sample 4 : **IM103**

Peak		Size [bp]	Conc. [pg/µl]	Molarity [pmol/l]	Observations
1	◀	35	125.00	5,411.3	Lower Marker
2		171	420.52	3,730.1	
3		342	19.73	87.3	
4		365	24.89	103.4	
5		378	16.02	64.1	
6		397	20.49	78.2	
7		486	5.07	15.8	
8		503	5.26	15.9	
9		533	5.23	14.9	
10		560	11.48	31.1	
11		576	19.64	51.7	
12		770	13.60	26.8	
13		812	5.68	10.6	
14		872	9.52	16.5	
15		1,001	7.03	10.6	
16		1,233	8.96	11.0	
17		1,434	14.81	15.6	
18		1,651	15.19	13.9	
19		1,790	10.90	9.2	
20		1,937	14.63	11.4	
21		2,121	18.03	12.9	
22		2,336	13.73	8.9	
23		2,751	14.01	7.7	
24		3,294	14.00	6.4	
25		4,126	24.81	9.1	
26		4,843	14.17	4.4	

Overall Results for sample 4 : **IM103**

Number of peaks found:	31
Noise:	0.4
Corr. Area 1:	487.8

SNU-C2B-6

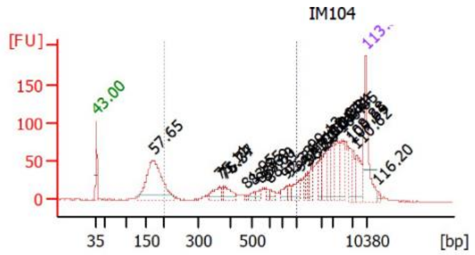

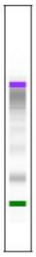

Peak table for sample 5 : **IM104**

Peak		Size [bp]	Conc. [pg/µl]	Molarity [pmol/l]	Observations
1	◀	35	125.00	5,411.3	Lower Marker
2		170	262.29	2,336.6	
3		368	27.08	111.5	
4		376	6.68	26.9	
5		384	23.54	92.9	
6		476	3.28	10.5	
7		511	9.09	27.0	
8		550	10.39	28.6	
9		578	8.18	21.4	
10		609	7.97	19.8	
11		787	13.77	26.5	
12		855	8.53	15.1	
13		1,123	23.27	31.4	
14		1,374	8.38	9.2	
15		1,499	9.91	10.0	
16		1,632	15.79	14.7	
17		1,953	17.74	13.8	
18		2,483	35.39	21.6	
19		2,763	19.02	10.4	
20		3,066	22.45	11.1	
21		3,563	28.12	12.0	
22		4,672	31.81	10.3	
23		6,430	17.13	4.0	
24		7,052	17.66	3.8	
25		8,017	30.98	5.9	

Overall Results for sample 5 : **IM104**

Number of peaks found:	25
Noise:	0.5
Corr. Area 1:	449.7

SNU-C2B-7

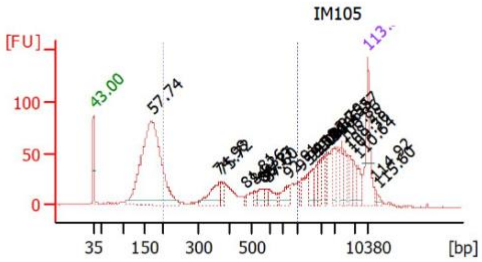

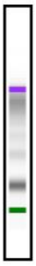

Peak table for sample 6 : **IM105**

Peak		Size [bp]	Conc. [pg/µl]	Molarity [pmol/l]	Observations
1	◀	35	125.00	5,411.3	Lower Marker
2		171	697.17	6,177.3	
3		366	59.14	244.5	
4		376	15.97	64.4	
5		473	6.12	19.6	
6		522	8.60	25.0	
7		559	21.13	57.2	
8		573	6.97	18.4	
9		591	20.83	53.4	
10		830	26.52	48.4	
11		1,120	9.17	12.4	
12		1,612	23.89	22.5	
13		1,747	12.83	11.1	
14		1,913	22.71	18.0	
15		2,316	14.51	9.5	
16		3,021	16.18	8.1	
17		4,454	16.11	5.5	
18		4,841	25.17	7.9	
19		6,118	17.17	4.3	
20		6,815	20.34	4.5	
21		8,036	18.91	3.6	
22	▶	10,380	75.00	10.9	Upper Marker

Overall Results for sample 6 : **IM105**

Number of peaks found:	22
Noise:	0.4
Corr. Area 1:	610.5

SNU-C2B-8

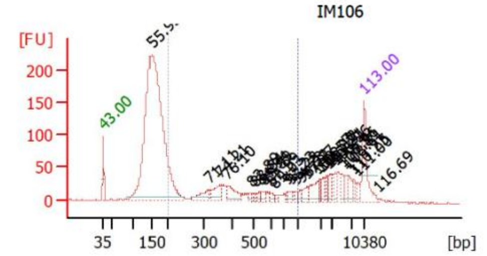

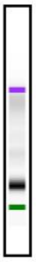

Peak table for sample 7 : **IM106**

Peak		Size [bp]	Conc. [pg/µl]	Molarity [pmol/l]	Observations
1	◀	35	125.00	5,411.3	Lower Marker
2		152	1,696.78	16,934.8	
3		318	44.16	210.5	
4		357	43.69	185.6	
5		381	49.90	198.7	
6		492	9.21	28.4	
7		511	6.49	19.2	
8		534	9.10	25.8	
9		554	11.80	32.3	
10		599	7.49	19.0	
11		632	5.68	13.6	
12		779	6.97	13.5	
13		853	10.60	18.8	
14		968	6.05	9.5	
15		1,164	10.60	13.8	
16		1,414	16.39	17.6	
17		1,865	32.14	26.1	
18		1,986	9.95	7.6	
19		2,228	11.21	7.6	
20		2,580	11.61	6.8	
21		2,804	14.70	7.9	
22		3,581	11.68	4.9	
23		4,919	11.91	3.7	
24		5,587	15.59	4.2	
25		6,374	18.93	4.5	
26		7,650	9.43	1.9	

Overall Results for sample 7 : **IM106**

Number of peaks found:	27
Noise:	0.5
Corr. Area 1:	677.9

SNU-C2B-9

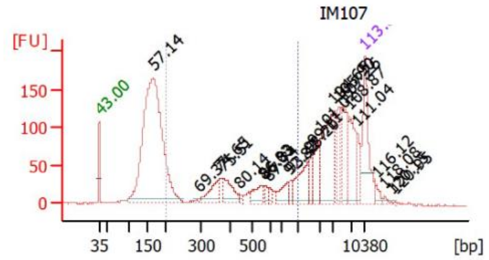

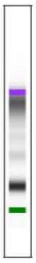

Peak table for sample 8 : **IM107**

Peak		Size [bp]	Conc. [pg/µl]	Molarity [pmol/l]	Observations
1	◀	35	125.00	5,411.3	Lower Marker
2		165	784.68	7,220.1	
3		297	2.71	13.8	
4		362	43.70	182.7	
5		373	47.17	191.5	
6		444	3.17	10.8	
7		557	24.91	67.7	
8		574	6.59	17.4	
9		594	5.59	14.3	
10		823	23.58	43.4	
11		868	10.09	17.6	
12		1,484	47.86	48.9	
13		1,630	18.26	17.0	
14		1,939	34.52	27.0	
15		3,097	91.63	44.8	
16		4,088	31.77	11.8	
17		5,159	42.04	12.3	
18		6,427	54.20	12.8	
19		8,429	21.15	3.8	
20	▶	10,380	75.00	10.9	Upper Marker

Overall Results for sample 8 : **IM107**

Number of peaks found:	22
Noise:	0.4
Corr. Area 1:	865.3

Panc-1-1

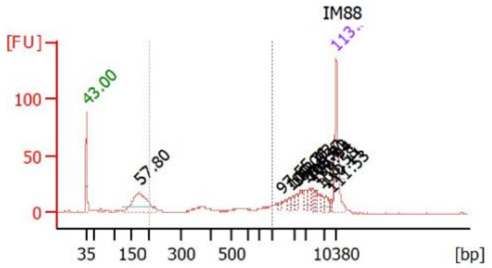

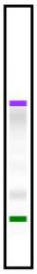

Peak table for sample 11 : **IM88**

Peak		Size [bp]	Conc. [pg/µl]	Molarity [pmol/l]	Observations
1	◀	35	125.00	5,411.3	Lower Marker
2		172	143.83	1,263.8	
3		1,380	5.50	6.0	
4		1,784	8.24	7.0	
5		1,890	6.49	5.2	
6		2,198	7.74	5.3	
7		2,938	8.27	4.3	
8		3,657	8.57	3.6	
9		4,298	10.29	3.6	
10		4,853	8.66	2.7	
11		6,134	8.33	2.1	
12		7,430	10.09	2.1	
13		8,877	3.32	0.6	
14	▶	10,380	75.00	10.9	Upper Marker

Overall Results for sample 11 : **IM88**

Number of peaks found:	12
Noise:	0.3
Corr. Area 1:	100.9

Panc-1-2

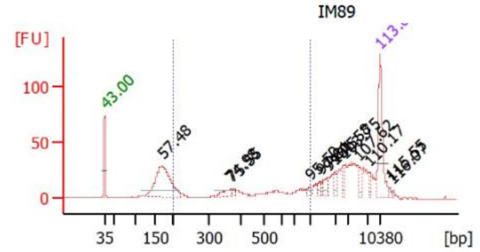

Overall Results for sample 1 : **IM89**

Number of peaks found:	12
Noise:	0.2
Corr. Area 1:	216.4

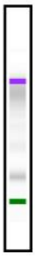

Peak table for sample 1 : **IM89**

Peak		Size [bp]	Conc. [pg/µl]	Molarity [pmol/l]	Observations
1	◄	35	125.00	5,411.3	Lower Marker
2		169	222.14	1,990.7	
3		367	19.82	81.8	
4		374	6.55	26.5	
5		1,043	6.67	9.7	
6		1,413	7.01	7.5	
7		1,722	13.46	11.8	
8		2,033	9.93	7.4	
9		2,728	9.77	5.4	
10		5,374	12.40	3.5	
11		7,491	6.74	1.4	
12	►	10,380	75.00	10.9	Upper Marker
13		12,980	0.00	0.0	
14		13,510	0.00	0.0	

Panc-1-3

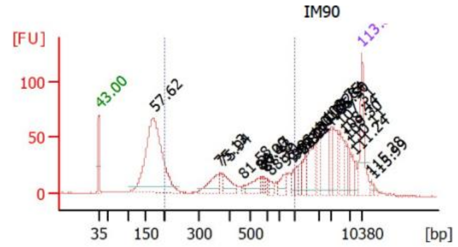

Overall Results for sample 2 : **IM90**

Number of peaks found:	25
Noise:	0.2
Corr. Area 1:	505.4

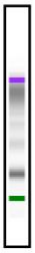

Peak table for sample 2 : **IM90**

Peak		Size [bp]	Conc. [pg/µl]	Molarity [pmol/l]	Observations
1	◄	35	125.00	5,411.3	Lower Marker
2		171	554.37	4,926.2	
3		369	58.47	240.0	
4		379	42.66	170.5	
5		470	4.18	13.5	
6		557	37.30	101.4	
7		567	9.92	26.5	
8		586	6.59	17.1	
9		622	10.12	24.6	
10		829	26.54	48.5	
11		973	11.36	17.7	
12		1,094	13.90	19.2	
13		1,284	15.76	18.6	
14		1,481	23.07	23.6	
15		1,823	43.10	35.8	
16		2,011	16.47	12.4	
17		2,725	61.61	34.3	
18		3,182	22.23	10.6	
19		3,705	40.42	16.5	
20		5,124	27.55	8.1	
21		6,020	18.21	4.6	
22		6,693	16.01	3.6	
23		7,425	18.17	3.7	
24		8,588	9.95	1.8	

G12A

NCI-H2009-1

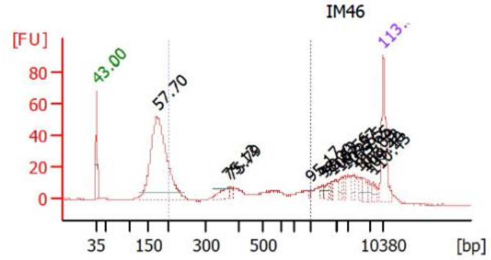

Overall Results for sample 5 : **IM46**

Number of peaks found:	14
Noise:	0.2
Corr. Area 1:	258.0

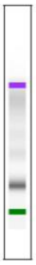

Peak table for sample 5 : **IM46**

Peak		Size [bp]	Conc. [pg/µl]	Molarity [pmol/l]	Observations
1	◄	35	125.00	5,411.3	Lower Marker
2		171	590.17	5,235.9	
3		368	25.25	104.0	
4		376	5.94	23.9	
5		993	3.46	5.3	
6		1,496	5.72	5.8	
7		1,698	8.64	7.7	
8		1,866	5.61	4.6	
9		2,385	7.66	4.9	
10		2,736	7.34	4.1	
11		3,936	7.72	3.0	
12		5,068	9.07	2.7	
13		6,038	11.86	3.0	
14		6,887	7.91	1.7	
15		7,865	9.92	1.9	
16	►	10,380	75.00	10.9	Upper Marker

NCI-H2009-2

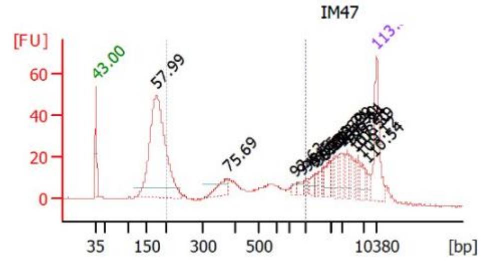

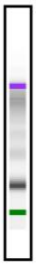

Peak table for sample 6 :			IM47	
Peak	Size [bp]	Conc. [pg/µl]	Molarity [pmol/l]	Observations
1	35	125.00	5,411.3	Lower Marker
2	174	617.31	5,381.1	
3	375	41.07	165.9	
4	804	6.26	11.8	
5	940	11.02	17.8	
6	1,047	8.18	11.8	
7	1,338	12.27	13.9	
8	1,484	8.62	8.8	
9	1,707	8.96	8.0	
10	1,990	20.51	15.6	
11	2,268	11.31	7.6	
12	2,524	14.89	8.9	
13	2,949	12.24	6.3	
14	3,906	13.18	5.1	
15	4,401	12.56	4.3	
16	5,389	12.26	3.4	
17	6,090	11.10	2.8	
18	6,667	18.95	4.3	
19	7,970	12.29	2.3	
20	10,380	75.00	10.9	Upper Marker

Overall Results for sample 6 : IM47

Number of peaks found:	18
Noise:	0.2
Corr. Area 1:	309.6

NCI-H2009-3

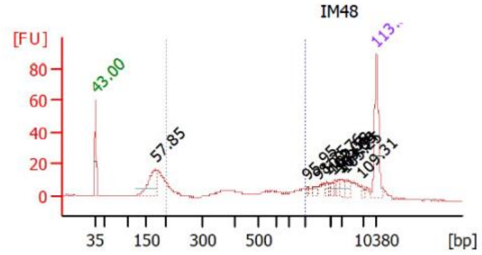

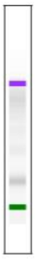

Peak table for sample 7 :			IM48	
Peak	Size [bp]	Conc. [pg/µl]	Molarity [pmol/l]	Observations
1	35	125.00	5,411.3	Lower Marker
2	172	104.46	917.8	
3	1,112	3.16	4.3	
4	1,488	8.12	8.3	
5	1,899	6.10	4.9	
6	2,216	6.06	4.1	
7	2,390	5.85	3.7	
8	2,737	6.99	3.9	
9	2,962	8.08	4.1	
10	3,623	13.67	5.7	
11	6,816	5.00	1.1	
12	10,380	75.00	10.9	Upper Marker

Overall Results for sample 7 : IM48

Number of peaks found:	10
Noise:	0.2
Corr. Area 1:	132.6

SW1116-1

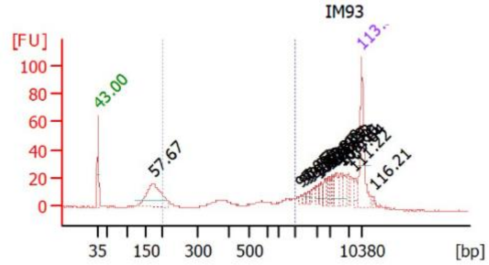

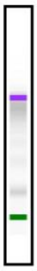

Peak table for sample 5 :			IM93	
Peak	Size [bp]	Conc. [pg/µl]	Molarity [pmol/l]	Observations
1	35	125.00	5,411.3	Lower Marker
2	171	164.92	1,460.5	
3	1,292	4.38	5.1	
4	1,438	4.14	4.4	
5	1,601	5.94	5.6	
6	1,732	4.77	4.2	
7	2,034	8.80	6.6	
8	2,279	10.65	7.1	
9	2,621	11.07	6.4	
10	2,964	8.46	4.3	
11	3,512	11.80	5.1	
12	4,354	15.25	5.3	
13	5,356	14.79	4.2	
14	5,797	12.30	3.2	
15	6,960	9.50	2.1	
16	8,560	10.35	1.8	
17	10,380	75.00	10.9	Upper Marker

Overall Results for sample 5 : IM93

Number of peaks found:	16
Noise:	0.2
Corr. Area 1:	162.7

SW1116-2

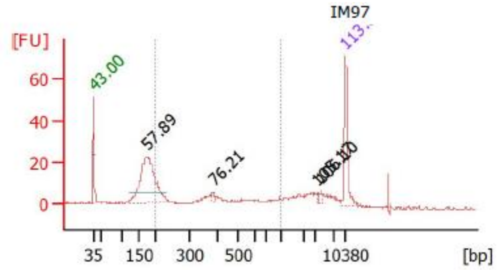

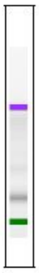

Peak table for sample 9 :			IM97	
Peak	Size [bp]	Conc. [pg/µl]	Molarity [pmol/l]	Observations
1	35	125.00	5,411.3	Lower Marker
2	173	373.97	3,267.3	
3	383	6.78	26.9	
4	3,447	3.90	1.7	
5	4,174	4.61	1.7	
6	10,380	75.00	10.9	Upper Marker

Overall Results for sample 9 : IM97

Number of peaks found:	4
Noise:	0.1
Corr. Area 1:	115.5

G13D

HCT-15-1

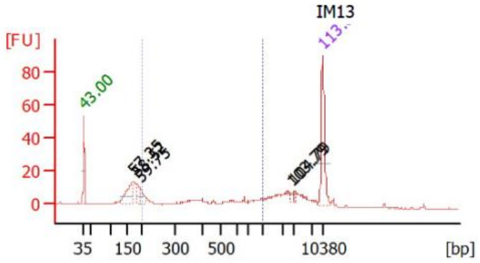

Overall Results for sample 10 : **IM13**

Number of peaks found:	5
Noise:	0.1
Corr. Area 1:	100.3

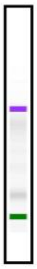

Peak table for sample 10 : **IM13**

Peak		Size [bp]	Conc. [pg/µl]	Molarity [pmol/l]	Observations
1	◀	35	125.00	5,411.3	Lower Marker
2		168	88.48	797.1	
3		181	30.46	255.5	
4		194	29.96	234.3	
5		2,739	4.22	2.3	
6		3,147	4.15	2.0	
7	▶	10,380	75.00	10.9	Upper Marker

HCT-15-2

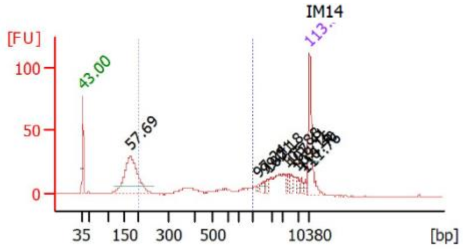

Overall Results for sample 11 : **IM14**

Number of peaks found:	9
Noise:	0.2
Corr. Area 1:	143.7

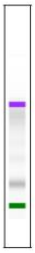

Peak table for sample 11 : **IM14**

Peak		Size [bp]	Conc. [pg/µl]	Molarity [pmol/l]	Observations
1	◀	35	125.00	5,411.3	Lower Marker
2		172	277.30	2,445.0	
3		1,320	3.19	3.7	
4		1,637	10.61	9.8	
5		1,792	6.56	5.5	
6		4,005	7.26	2.7	

HCT-15-3

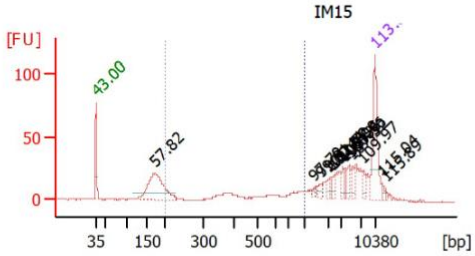

Overall Results for sample 1 : **IM15**

Number of peaks found:	13
Noise:	0.2
Corr. Area 1:	148.0

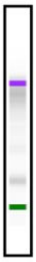

Peak table for sample 1 : **IM15**

Peak		Size [bp]	Conc. [pg/µl]	Molarity [pmol/l]	Observations
1	◀	35	125.00	5,411.3	Lower Marker
2		172	175.71	1,547.8	
3		1,400	4.38	4.7	
4		1,663	6.90	6.3	
5		2,023	6.40	4.8	
6		2,208	6.33	4.3	
7		2,438	6.80	4.2	
8		3,422	9.75	4.3	
9		3,864	8.13	3.2	
10		4,639	13.93	4.5	
11		5,229	14.47	4.2	
12		7,375	14.59	3.0	
13	▶	10,380	75.00	10.9	Upper Marker
14		12,399	0.00	0.0	
15		13,245	0.00	0.0	

MDA-MB-231-1

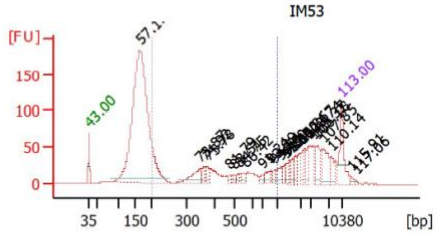

Overall Results for sample 1 : **IM53**

Number of peaks found:	24
Noise:	0.2
Corr. Area 1:	641.8

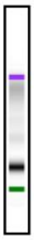

Peak table for sample 1 : **IM53**

Peak		Size [bp]	Conc. [pg/µl]	Molarity [pmol/l]	Observations
1	◀	35	125.00	5,411.3	Lower Marker
2		163	1,656.23	15,386.5	
3		350	64.15	277.5	
4		363	22.38	93.3	
5		374	18.98	76.8	
6		470	6.52	21.0	
7		500	11.93	36.2	
8		526	11.20	32.3	
9		561	18.54	50.1	
10		698	11.80	25.6	
11		853	24.16	42.9	
12		930	10.42	17.0	
13		1,152	21.57	28.4	
14		1,317	14.56	16.7	
15		1,452	14.49	15.1	
16		1,665	15.88	14.5	
17		1,823	18.08	15.0	
18		2,240	15.11	10.2	
19		2,749	24.04	13.2	
20		3,669	19.80	8.2	
21		4,310	35.41	12.4	
22		5,630	49.03	13.2	
23		7,551	20.84	4.2	
24	▶	10,380	75.00	10.9	Upper Marker

MDA-MB-231-2

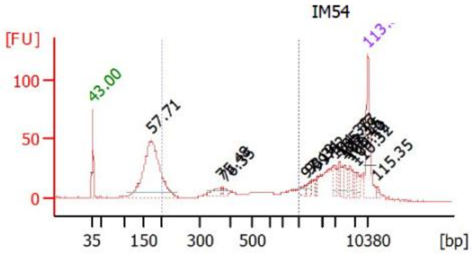

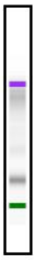

Peak table for sample 2 : **IM54**

Peak		Size [bp]	Conc. [pg/µl]	Molarity [pmol/l]	Observations
1	◀	35	125.00	5,411.3	Lower Marker
2		169	464.01	4,153.3	
3		371	23.89	97.7	
4		381	6.77	26.9	
5		1,299	8.71	10.2	
6		1,489	7.11	7.2	
7		1,751	5.70	4.9	
8		2,954	11.03	5.7	
9		3,985	12.27	4.7	
10		4,477	11.81	4.0	
11		5,083	10.13	3.0	
12		6,068	13.34	3.3	
13		6,636	9.55	2.2	
14		7,729	8.24	1.6	
15	▶	10,380	75.00	10.9	Upper Marker
16		12,700	0.00	0.0	

Overall Results for sample 2 : **IM54**

Number of peaks found:	14
Noise:	0.3
Corr. Area 1:	243.1

MDA-MB-231-3

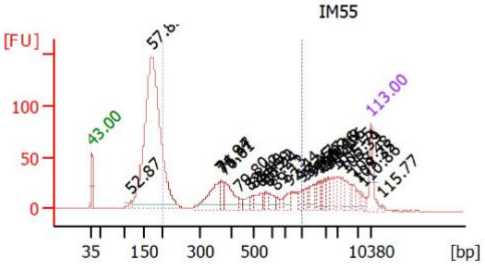

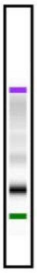

Peak table for sample 3 : **IM55**

Peak		Size [bp]	Conc. [pg/µl]	Molarity [pmol/l]	Observations
1	◀	35	125.00	5,411.3	Lower Marker
2		119	21.63	276.0	
3		171	1,945.98	17,291.6	
4		364	165.83	690.9	
5		370	29.41	120.3	
6		377	120.67	484.7	
7		435	14.12	49.2	
8		481	35.00	110.3	
9		498	17.35	52.8	
10		539	41.03	115.3	
11		557	14.57	39.6	
12		578	37.22	97.5	
13		629	11.79	28.4	
14		783	30.04	58.1	
15		1,011	21.25	31.8	
16		1,180	25.83	33.2	
17		1,510	29.86	30.0	
18		1,751	22.51	19.5	
19		1,840	19.48	16.0	
20		1,960	25.04	19.4	
21		2,237	21.63	14.7	
22		2,881	21.16	11.1	
23		3,940	18.36	7.1	
24		4,478	34.56	11.7	
25		6,130	21.51	5.3	
26		6,822	12.94	2.9	

Overall Results for sample 3 : **IM55**

Number of peaks found:	27
Noise:	0.3
Corr. Area 1:	687.1

MDA-MB-231-4

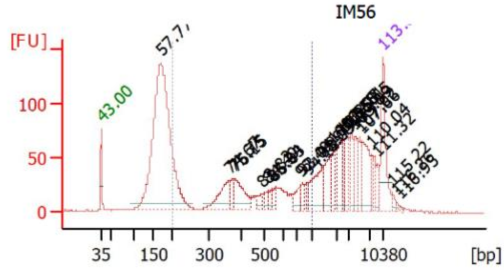

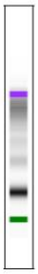

Peak table for sample 4 : **IM56**

Peak		Size [bp]	Conc. [pg/µl]	Molarity [pmol/l]	Observations
1	◀	35	125.00	5,411.3	Lower Marker
2		170	975.80	8,700.8	
3		360	67.16	282.5	
4		374	24.43	99.0	
5		379	66.85	267.3	
6		488	12.81	39.8	
7		518	11.92	34.8	
8		538	11.56	32.5	
9		553	9.44	25.9	
10		780	26.10	50.7	
11		828	10.84	19.8	
12		911	9.20	15.3	
13		1,438	63.20	66.6	
14		1,749	39.23	34.0	
15		1,871	20.97	17.0	
16		2,372	29.68	19.0	
17		2,531	20.08	12.0	
18		2,771	29.92	16.4	
19		3,495	21.30	9.2	
20		4,081	36.30	13.5	
21		4,979	18.38	5.6	
22		5,486	62.17	17.2	
23		7,451	14.09	2.9	
24		8,720	14.75	2.6	
25	▶	10,380	75.00	10.9	Upper Marker

Overall Results for sample 4 : **IM56**

Number of peaks found:	26
Noise:	0.3
Corr. Area 1:	814.3

MDA-MB-231-5

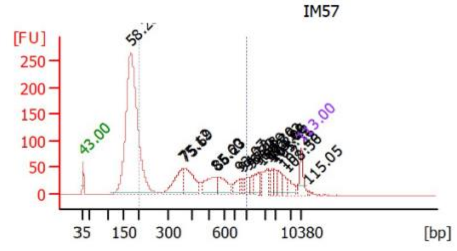

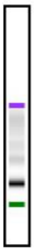

Peak table for sample 10 : IM57

Peak		Size [bp]	Conc. [pg/µl]	Molarity [pmol/l]	Observations
1	◄	35	125.00	5,411.3	Lower Marker
2		175	2,702.84	23,466.3	
3		364	247.49	1,030.1	
4		371	249.05	1,016.5	
5		542	157.69	441.2	
6		555	139.77	381.8	
7		825	59.91	110.1	
8		917	20.69	34.2	
9		1,125	50.59	68.1	
10		1,304	32.28	37.5	
11		1,587	63.14	60.3	
12		1,804	30.19	25.4	
13		2,339	26.01	16.8	
14		2,694	33.93	19.1	
15		2,844	37.38	19.9	
16		3,688	63.31	26.0	
17		5,273	29.71	8.5	
18		6,109	48.24	12.0	
19	►	10,380	75.00	10.9	Upper Marker
20		12,487	0.00	0.0	

Overall Results for sample 10 : IM57

Number of peaks found: 18
Noise: 0.2
Corr. Area 1: 1,269.9

Q61H

NCI-H460-1

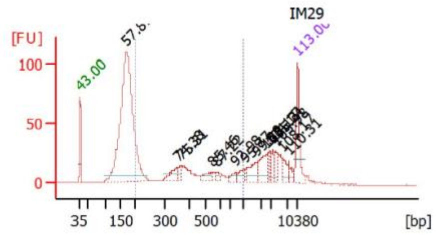

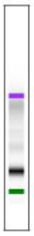

Peak table for sample 7 : IM29

Peak		Size [bp]	Conc. [pg/µl]	Molarity [pmol/l]	Observations
1	◄	35	125.00	5,411.3	Lower Marker
2		170	1,147.00	10,235.4	
3		355	41.64	177.9	
4		366	15.02	62.1	
5		538	23.53	66.2	
6		576	10.13	26.7	
7		818	13.00	24.1	
8		1,055	18.97	27.2	
9		1,736	38.71	33.8	
10		2,538	43.08	25.7	
11		2,880	12.72	6.7	
12		3,522	17.53	7.5	
13		4,116	12.59	4.6	
14		5,811	23.89	6.2	
15		7,618	13.01	2.6	
16	►	10,380	75.00	10.9	Upper Marker

Overall Results for sample 7 : IM29

Number of peaks found: 14
Noise: 0.1
Corr. Area 1: 398.1

NCI-H460-2

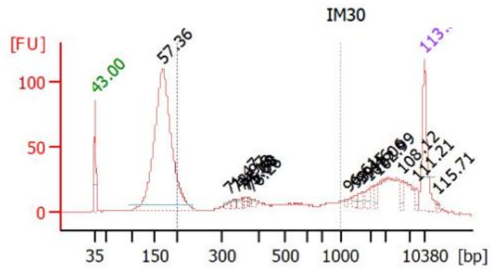

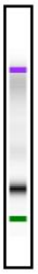

Peak table for sample 2 : IM30

Peak		Size [bp]	Conc. [pg/µl]	Molarity [pmol/l]	Observations
1	◄	35	125.00	5,411.3	Lower Marker
2		167	879.36	7,984.3	
3		321	7.94	37.5	
4		340	10.80	48.1	
5		355	10.07	42.9	
6		366	6.09	25.2	
7		373	4.95	20.1	
8		382	6.76	26.8	
9		1,219	3.41	4.2	
10		1,517	6.54	6.5	
11		1,681	6.59	5.9	
12		1,940	7.82	6.1	
13		2,508	7.35	4.4	
14		5,817	9.57	2.5	
15		8,585	7.40	1.3	
16	►	10,380	75.00	10.9	Upper Marker
17		13,097	0.00	0.0	

Overall Results for sample 2 : IM30

Number of peaks found: 15
Noise: 0.2
Corr. Area 1: 350.7

NCI-H460-3

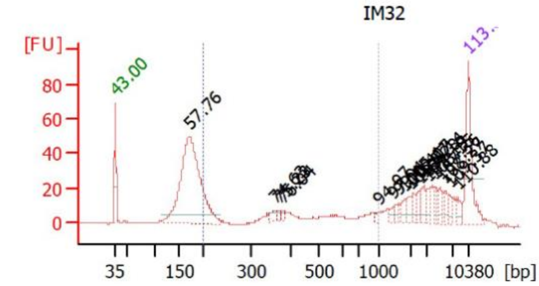

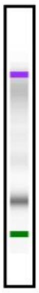

Peak table for sample 4 : IM32

Peak		Size [bp]	Conc. [pg/µl]	Molarity [pmol/l]	Observations
1	◀	35	125.00	5,411.3	Lower Marker
2		171	493.63	4,372.2	
3		361	10.05	42.2	
4		369	6.46	26.6	
5		379	5.36	21.4	
6		978	2.77	4.3	
7		1,435	6.08	6.4	
8		1,631	8.38	7.8	
9		1,835	10.93	9.0	
10		2,045	11.11	8.2	
11		2,209	7.79	5.3	
12		2,601	10.85	6.3	
13		2,879	14.25	7.5	
14		3,534	11.39	4.9	
15		4,281	10.25	3.6	
16		5,225	13.01	3.8	
17		5,933	13.08	3.3	
18		6,798	6.99	1.6	
19		8,257	9.24	1.7	
20	▶	10,380	75.00	10.9	Upper Marker

Overall Results for sample 4 : IM32

Number of peaks found: 18
Noise: 0.2
Corr. Area 1: 216.5

NCI-H460-4

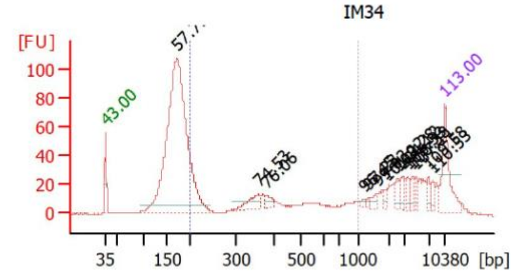

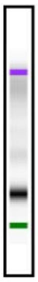

Peak table for sample 6 : IM34

Peak		Size [bp]	Conc. [pg/µl]	Molarity [pmol/l]	Observations
1	◀	35	125.00	5,411.3	Lower Marker
2		171	1,088.81	9,668.0	
3		360	39.28	165.4	
4		379	16.98	67.9	
5		1,192	3.53	4.5	
6		1,320	3.61	4.1	
7		1,610	11.02	10.4	
8		1,933	6.32	4.9	
9		2,600	13.58	7.9	
10		2,873	14.27	7.5	
11		3,638	11.54	4.8	
12		4,458	12.47	4.2	
13		5,033	9.86	3.0	
14		6,962	9.87	2.1	
15		7,901	13.17	2.5	
16	▶	10,380	75.00	10.9	Upper Marker

Overall Results for sample 6 : IM34

Number of peaks found: 14
Noise: 0.3
Corr. Area 1: 389.4

NCI-H460-5

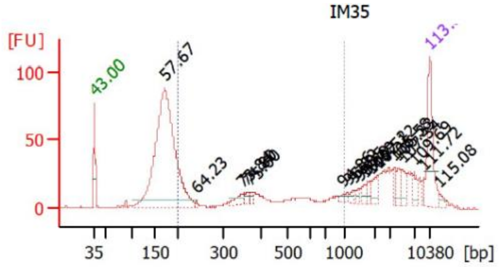

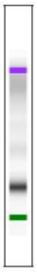

Peak table for sample 7 : IM35

Peak		Size [bp]	Conc. [pg/µl]	Molarity [pmol/l]	Observations
1	◀	35	125.00	5,411.3	Lower Marker
2		170	748.00	6,659.3	
3		240	3.29	20.8	
4		351	16.40	70.8	
5		363	9.87	41.2	
6		373	8.71	35.3	
7		970	4.27	6.7	
8		1,090	3.40	4.7	
9		1,289	6.58	7.7	
10		1,506	7.31	7.4	
11		1,670	9.03	8.2	
12		1,852	9.51	7.8	
13		2,223	15.29	10.4	
14		3,838	11.17	4.4	
15		4,421	15.09	5.2	
16		5,213	16.71	4.9	
17		7,060	12.93	2.8	
18		9,095	7.20	1.2	
19	▶	10,380	75.00	10.9	Upper Marker
20		12,469	0.00	0.0	

Overall Results for sample 7 : IM35

Number of peaks found: 18
Noise: 0.3
Corr. Area 1: 345.6
